# Blocking of stromal interaction molecule 1 expression influence cell proliferation and promote cell apoptosis in vitro and inhibit tumor growth in vivo in head and neck squamous cell carcinoma

**DOI:** 10.1371/journal.pone.0177484

**Published:** 2017-05-11

**Authors:** Ping Li, Xue-yan Bian, Qing Chen, Xiao-feng Yao, Xu-dong Wang, Wen-chao Zhang, Ying-jie Tao, Rui Jin, Lun Zhang

**Affiliations:** 1 Departments of Maxillofacial and Otorhinolaryngology Oncology, Tianjin Medical University Cancer Institute and Hospital, Tianjin, the People's Republic of China; 2 National Clinical Research Center of Cancer, Key Laboratory of Cancer Prevention and Therapy, Tianjin, the People's Republic of China; 3 Cardiopulmonary Function Laboratory, Tianjin Medical University Cancer Institute and Hospital, Tianjin, the People's Republic of China; University of South Alabama Mitchell Cancer Institute, UNITED STATES

## Abstract

Calcium signal plays an important role in a variety of cancer cell metabolism, but knowledge on its role in head and neck squamous cell carcinoma (HNSCC) is limited. Store-operated calcium entry (SOCE) is the principal Ca^2+^ entry mechanism that maintains calcium concentration and produces calcium signal in non-excitable cells. SOCE is triggered by stromal interaction molecule 1 (STIM1), which is located in endoplasmic reticulum (ER) as Ca^2+^ sensor. Although, many studies demonstrated that STIM1 and SOCE play important functions in the regulation of many cancer progressions, their clinical relevance in HNSCC remains unclear. In this study, STIM1 expression levels notably increased in 89% HNSCC tissues compared with those in adjacent normal tissues. Meanwhile, this overexpression was close associated with tumor size but not with neck lymph node metastasis. Thus, this study mainly focuses on STIM1 function in HNSCC tumor growth. Three HNSCC cell lines, namely, TSCCA (oral cancer cell line) and Hep2 (laryngeal cell line) with high STIM1 expression levels and Tb3.1 (oral cancer cell line) with STIM1 expression level lower than previous two cell lines, were selected for in vitro study. Downregulated STIM1 expression levels in TSCCA and Hep2 arrested cells in G0/G1 stages, promoted cell apoptosis, and inhibited cell proliferation. By contrast, upregulated STIM1 expression in Tb3.1 inhibited cell apoptosis and promoted cell proliferation. Induced by thapsigargin (TG), ER stress was amplified when STIM1 expression was downregulated but was attenuated as STIM1 expression was upregulated. Furthermore, TSCCA cell xenograft models confirmed that STIM1 could promote HNSCC tumor growth in vivo. The present study provides new insight into HNSCC molecular mechanism and potential therapeutic target through targeting SOCE-dependent process. However, whether STIM1 participates in HNSCC metastasis requires further study.

## Introduction

Head and neck squamous cell carcinoma (HNSCC) is the six more common cancer in worldwide, the estimated new cases has achieved 4% in males [1]. Factors, such as tobacco [2], alcohol[3]consumption, exposure to HPV[4], physical and inflammatory stimulation[5], are identified as the dominant causes of HNSCC development. Despite advances in combined treatment, the overall 5-year survival rate for HNSCC remains at 50–55% over the past several decades [6]. Moreover, the overall 5-year survival rate for HNSCC in some Chinese undeveloped areas is only 20%–45% because of low economic level and limited medical technology in these areas. Therefore, in-depth understanding of the molecular mechanism, which is related to tumorigenesis and progression, is essential to improve the treatment effects for HNSCC patients.

Calcium (Ca^2+^) signal is an important second signal in cellular metabolism[7]. Furthermore, many studies have demonstrated that intracellular Ca^2+^ signal participate in various cancer progression, such as proliferation, apoptosis and migration[7–10]. Store-operated calcium entry (SOCE) was a channel mainly involved in the maintenance of intracellular calcium homeostasis through extracellular Ca^2+^ influx pathway in non-excitable cells, such as epithelial cells[11]. Stromal interaction molecule 1 (STIM1) is a Ca^2+^ sensor located at the endoplasmic reticulum (ER) and is a key component of SOCE [12, 13]. STIM1 is required for the development and function of regulatory T cells, and STIM1 deficiency causes several autoimmune diseases and myopathy in human subjects and mouse models [14]. Several studies have demonstrated that STIM1-mediated SOCE dysregulation is involved in tumor development and progression [15, 16]. Thus, inhibiting STIM1-dependent Ca^2+^ signaling by specifically targeting STIM1 activation and translocation in cancer cells is a potential target for cancer therapy for breast [17], cervical[18], colon[19, 20], prostate[21] and hepatic cancer [22]. However, few studies have reported the expression of STIM1 and its relevant biological functions in HNSCC.

In this study, attention was focused on the role of STIM1 in HNSCC from clinical significance to mice model verification. Results indicated that STIM1 is important for Ca^2+^ signal, which is necessary to maintain the calcium hemostasis, improve cell anti-apoptosis ability, and promote cell growth in HNSCC cells. Our study may provide a new insight into mechanism investigation and molecular therapy of HNSCC.

## Materials and methods

### 2.1 Patients and tissues specimen

All tissue samples from patients who had undergone wide excision with neck lymph node dissection for HNSCC were collected in Tianjin Medical University Cancer Institute and Hospital (TJMUCIH) from 2010 to 2011. A total of 56 formalin fixed paraffin embedded (FFPE) tissue including tumor and adjacent normal tissue which identified by three experienced pathologists were collected for immunohistochemistry (IHC). All patients did not exhibit distant organs metastasis before operation. Meanwhile, another 8 fresh paired specimens including tumor tissues and the adjacent normal tissues from same HNSCC patients were used for western blotting (WB) detection. The collection of surgical specimens was approved by the institutional review board of TJMUCIH.

### 2.2 Cell cultures, transfection, and regents

Hep2 (laryngeal cancer cell line) and Cal27 (oral cancer cell line) were purchased from ATCC (American type culture collection). TSCCA, Tca8113 (oral cancer cell line) were purchased from the Institute of Basic Medical Sciences, Chinese Academy of Medical Sciences(Peking, China), Tb3.1 (oral cancer cell line) was an kindly gift from the Ninth People’s Hospital Shanghai Jiao Tong University. TSCCA and Hep2 cell lines were cultured in MEM medium (Gibco, USA), Tb3.1 and TCA8113 cell lines were cultured in RPMI-1640 medium (Gibco, USA), and Cal27 was cultured in DMEM medium (Gibco, USA) supplemented with 10% fetal bovine serum (Gibco, USA). All cell lines were cultured at 37°C in a humidified 5% CO2 incubator. TSCCA and Hep2 cells were transfected with 50 nM of small interfering RNA (siRNA) against STIM1 (Abnova, China) using lipo3000 (Invitrogen, USA) according to the manufacture’s instructions. Tb3.1 cells were transfected with GV144-STIM1 to upregulate STIM1 expression (Genechem, China). SKF96365 (ab120280), an inhibitor for store-operated Ca^2+^ entry, was obtained from Abcam. Thapsigargin (TG), which used to induce SOCE or ER stress, was obtained from Sigma (St. Louis, USA). Fura-2 AM, which is high affinity and intracellular calcium indicator, was purchased from Thermo Fisher Scientific. DMEM and RPMI-1640 medium with calcium free were purchased from Gibco. EGTA, which used for chelating calcium ion, was purchased from Thomas Scientific. Cell cycle and apoptosis were performed at 48 hours after transfection. Cell proliferation was performed 72 hours after transfection.

### 2.3 IHC staining

STIM1, Cyclin D1, P21, BAX, Bcl2, caspase3 and caspase12 expression in histological level were detected using IHC staining in FFPE samples. The sections were incubated with primary antibodies overnight at 4°C, followed by a biotin-labeled secondary antibody (1:100 dilutions) for 1h at room temperature. Sections were detected using DAB kit and counterstained with hematoxylin and visualized using light microscopy.

### 2.4 Western blot analysis

Cells and frozen tissues were treated with RIPA buffer (Sigma, USA). Equal amounts of protein were subjected to sodium dodecyl sulfate polyacrylamide gelelectro phoresis (SDS-PAGE) followed by transfer to PVDF membranes (Millipore, USA). The membranes were blocked with 5% fat-free milk (BD, USA) for 1.5 h at room temperature and incubated with primary antibodies at 4°C overnight. The membranes were then incubated with secondary antibodies for 1 h at room temperature. The following antibodies were used in this study: mouse anti-STIM1 mAb (Abcam, ab57834), rat anti-caspase12 (Millipore, MABC555), rabbit anti-caspase3 (CST, #9662), rabbit anti-cyclin D1 (CST, #2922), rabbit anti-P21 (CST, #2947), mouse anti-Bcl2 (ZSGB-BIO, ZS-7382), rabbit anti-BAX (CST, #5023). GAPDH and β-actin (ZSGB-BIO, China) were used as internal control.

### 2.5 Real-time (RT) PCR analysis

Total RNA was extracted from HNSCC cell lines using a TRIzol reagent (Invitrogen, USA). PCR primers were purchased from AuGCT (Beijing, China). Total RNA aliquots (1μg) were reverse transcribed at 25°C for 10 min, 42°C for 60 min and 70°C for 15 min using a reverse transcription system (Promega, China). Reaction mixture aliquots (cDNA, 1μl) were used as templates for RT-PCR and subjected to qPCR Master Mix (Promega, China). PCR cycling conditions were 95°C for 2 min and 40 cycles of 95°C for 15 s, and 60°C for 60 s followed by a final melting curve program. GAPDH RNA was used as loading control. Each sample was analyzed in triplicate, and the mean values were used for quantization. The primers for STIM1 and GAPDH were as follows: forward: 5′-TGTGGAGCTGCCTCAGTATG-3′ and reverse 5′-CTTCAGCACAGTCCCTGTCA-3′ (STIM1); forward 5′ TTCGTCATGGCTGTGAACCA-3′ and reverse 5′-CAGTGATGCGCATGGACTGT -3′ (GAPDH)

### 2.6 Measurement of [Ca^2+^]_i_ in HNSCC cell lines

Ratiometric imaging of intracellular Ca^2+^ using furo-2 was similar to a previous study [23]. In brief, Ca^2+^ measurements were performed under a Leica DMI 6000B fluorescence microscope controlled by Slide Book Software. When the cell was placed under the microscope, a 20-min resting period was sustained to allow the cells to recover to a stable state for repetitive calcium responses. The calcium response of the cells was recorded with CCD for 660 seconds (s) and the first 60 s for baseline. The fluorescence intensity of the calcium concentration for each cell was later normalized by its corresponding baseline. The first 120 s was calcium-free as the following 440 s changed into 2mmol/L calcium concentration in solution. TG was added in 100 s.

Fluorescence emission at 505 nm was monitored while it was alternating between 340 and 380 nm excitation wavelengths at 0.67 Hz. Intracellular Ca^2+^ measurements were shown as 340/380 nm emission ratios obtained from the groups (35 to 45) of single cells. Measurements shown are representative of a minimum of three independent experiments.

### 2.7 Apoptosis assays, proliferation curves and cell cycle distribution

Cell apoptosis and cell cycle distribution were determined by FACS. Cells were seeded at 10cm dishes and harvested after 48 hours by trypsinization, centrifuged, washed with PBS. Staining with Annexin V and Propidium iodide was according to the manufacturer's instructions and measured by flow cytometry on the FACS Calibur (BD Biosciences, San Jose, CA) [24].

Cells were washed with PBS and resuspended in 1ml of PBS. After following incubation for 1h in the dark at room temperature with RNase and PI, cells were analyzed by flow cytometry using a FACS Calibur flow cytometer (BD Biosciences, San Jose, CA) at 48h after transduction[25, 26].

1×10^5^ cells were seeded in 6 well culture-plates. The extracellular calcium concentration were 0, 2 and 4mmol/l respectively. After 24 hours cell culture, cells were trypsinized for cell counting using Coulter counter, then resuspended and reseeded in dishes for 48hrs and repeated the above experiments. Independent experiment for each group ≥3.

### 2.8 Animal experiments

Nude mouse subcutaneous TSCCA xenograft model was performed as described previously[24]. The mice were randomized into two groups when tumors reached 5 mm in length. One group was treated with PBS as negative control and the other groups were treated with STIM1 siRNA by local subcutaneous injection (SI). Tumor size was measured using a micro caliper (once every 3 days for 27 days), and tumor volume was calculated using the following formula: Volume = length×width^2^/2. At the end of the observation period, the mice bearing xenograft were sacrificed by pulling the necks. After the experiment, the animals were be placed in a garbage bag and sent to the school animal center. The tumor tissues were extracted for formalin fixation and preparation of paraffin-embedded sections. The animal experiments were performed according to the ethical guidelines and approved by the institutional ethical committee of TJMUCIH.

### 2.9 Statistical analysis

Image J (version 2x 2.1.4.7, National Institutes of Health, USA) was used for WB data collection. Origin Software (version 9.0, USA) was used for data analysis. Statistical comparisons between two groups of data were evaluated using Student’s t-test. A *P* value of < 0.05 was considered significant. Data were presented as mean ± SEM.

## Results

### 3.1 STIM1 was highly expressed in HNSCC tumor tissues and associated with clinical outcomes

**Fig 1:**
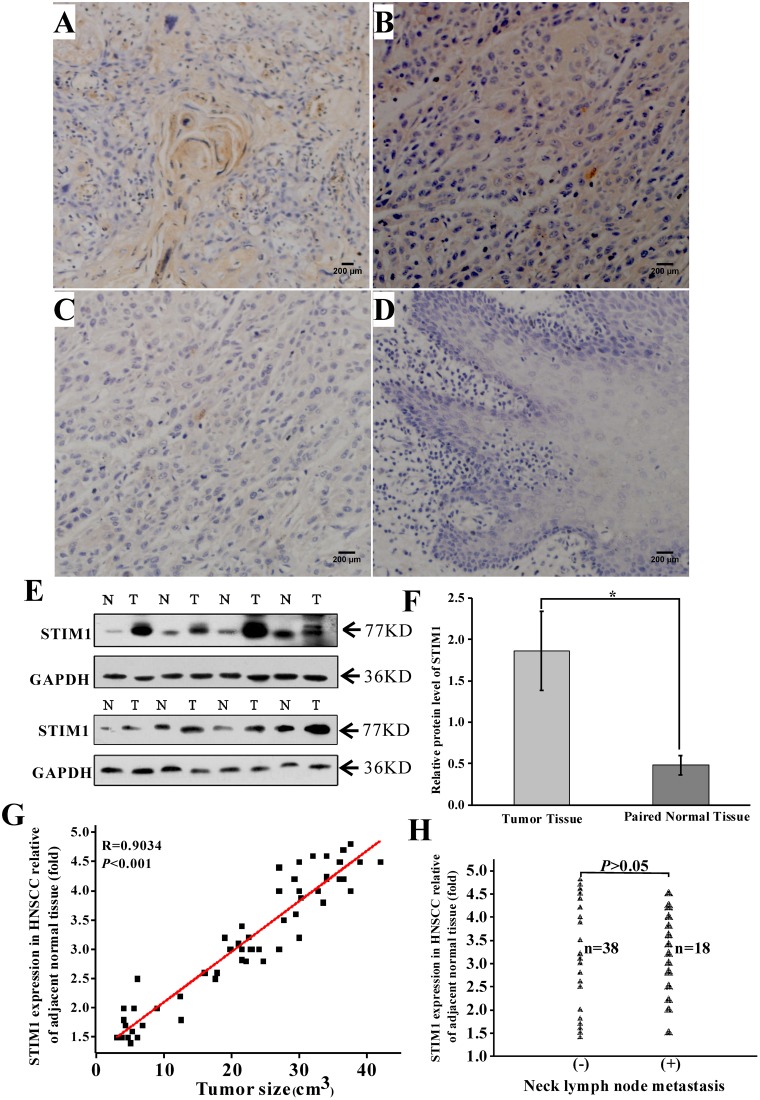
Overexpression of STIM1 in HNSCC tumor tissue was closely related to tumor size but not to neck node metastasis. STIM1 expression was detected by immunohistochemistry using monoclonal mouse anti-STIM1 antibody. STIM1 immunoreactivity was visible in the cytoplasm but not in the cell nucleus or on the membranes. (A) Paraffin-embedded sections of tongue squamous carcinoma tissue showed overexpression of STIM1 (Yellow staining). (B) Epiglottis squamous carcinoma tissue showed overexpression of STIM1 (Yellow staining). (C) Paired adjacent normal tissue of tongue showed relatively lower expression level of STIM1. (D) Paired adjacent normal tissue of epiglottis showed relatively lower expression level of STIM1. (E) Protein levels of STIM1 in 56 pairs of HNSCC cancer samples were analyzed by immunoblotting. Results from eight typical samples are shown here. (F) Quantitative analyses of STIM1 immunoblotting. The mean grays were collected through Image J software analysis. STIM1 expression in tumor tissue compared with GAPDH was significantly higher than in adjacent normal tissue. *p<0.05. (G) Association between STIM1 expression and tumor size in HNSCC tissues (*n* = 56). The value of linear relevance was 0.9034 between them. STIM1 expression in tumor tissue showed higher expression level than adjacent normal tissue in 50 cases (89%). (H) STIM1 expression in tumor tissue had no statistical difference between neck lymph node metastasis positive and negative groups (*p>*0.05).

IHC results showed that STIM1 expression levels in tongue (Fig 1A) and epiglotic (Fig 1C) tumor tissues were higher compared with that of adjacent normal tissue (Fig 1B and 1D). WB test in 16 paired tissues further verified IHC results (Fig 1E and 1F; *p<*0.05). Overexpression of STIM1 in tumor tissue was closely related to tumor size (Fig 1F; linear fit, *R* = 0.9034, *p*< 0.001) but not to neck lymph node metastasis. (Fig 1G; *p*> 0.05).

### 3.2 STIM1 expression showed difference in HNSCC cell lines and its downregulated or upregulated expression could decrease or increase SOCE in HNSCC cell lines

**Fig 2:**
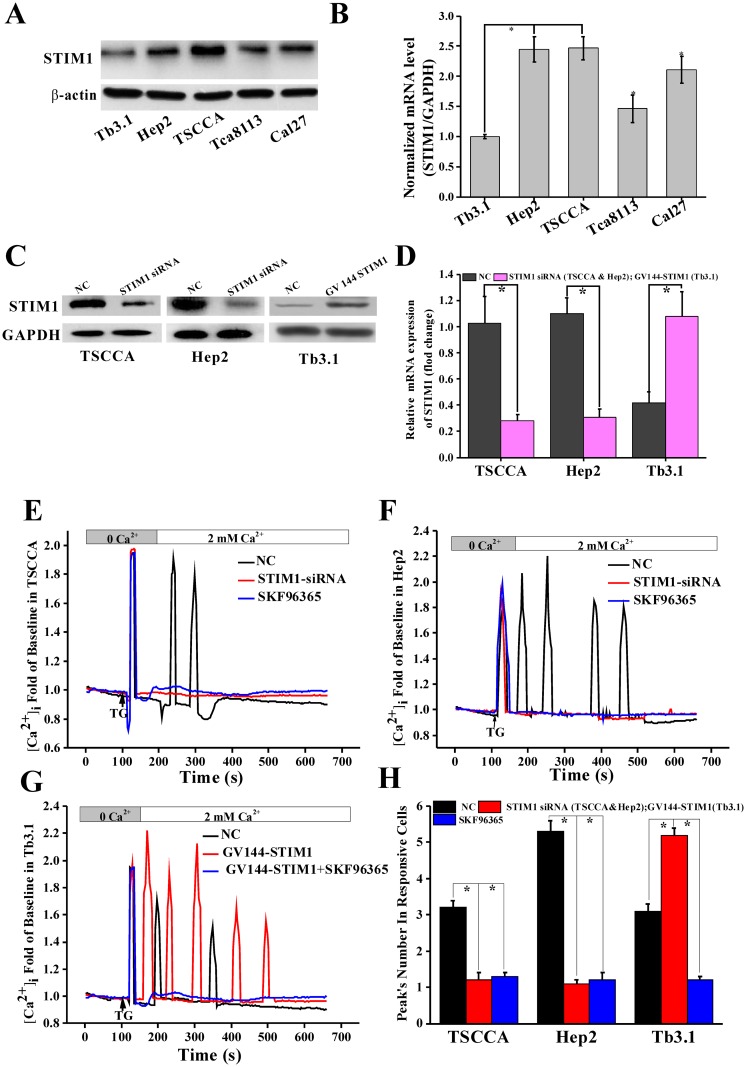
Change STIM1 expression or function influence SOCE in HNSCC cell lines. (A)Protein level of STIM1 showed different expression in five available HNSCC cell lines. TSCCA and Hep2 were the highest but lowest in Tb3.1. (B) Results of STIM1 expression in mRNA showed higher expression level in TSCCA and Hep2 but lower in Tb3.1. (C) Downregulated STIM1 in TSCCA and Hep2 by transfected with STIM1-siRNA and upregulated STIM1 expression in Tb3.1 by transfecting with GV-144 STIM1. The transfection efficiency was confirmed by WB. (D) Above transfection efficiency was further confirmed by RT-PCR detection* *p*< 0.05. (E) TG-induced SOCE in TSCCA. SOCE was found in normal group but not in STIM1-siRNA and added SKF96365 groups. (F) TG-induced SOCE in Hep2. SOCE was found in normal group but not in STIM1-siRNA and added SKF96365 groups. (G) TG-induced SOCE in Tb3.1. SOCE was found in normal group and GV144-STIM1 groups but not in GV144-STIM1+SKF96365 groups. (H) Mean number of calcium response peaks, which mainly reflect cell SOCE ability, was significantly decreased in STIM1 siRNA and add SKF96365 groups in TSCCA and Hep2 but significantly increased in GV144 STIM1 group for Tb3.1. *p< 0.05 versus other two groups.

The STIM1 expression levels in five available HNSCC cell lines, including TSCCA, Hep2, Tb3.1, Tca8113, and Cal27, were detected using RT-PCR and WB. The results showed that STIM1 expression was high in TSCCA and Hep2 but low in Tb3.1 (*p*< 0.05; Fig 2A). Thus, TSCCA, Hep2, and Tb3.1 cell lines were selected in this experiment.

For the identification of the function of STIM1 in HNSCC, TSCCA and Hep2 were transfected with STIM1-siRNA to downregulate STIM1 expression, and Tb3.1 was transfected with GV144-STIM1 to upregulate STIM1 expression. The transfection efficiency was confirmed using WB and RT-PCR after 48 h (Fig 2C).

TSCCA, Hep2, and Tb3.1 cells were used as normal control (NC) groups, and cells transfected with STIM1 siRNA, GV144-STIM1 and added with SKF96365 (50μM) were used as experiment groups. SOCE was induced by adding 1μM TG in NC and experiment groups (S1 Fig). Results showed that SOCE was diminished in STIM1 siRNA groups in TSCCA (Fig 2E) and Hep2 (Fig 2F) but strengthened in GV144-STIM1 groups in Tb3.1 (Fig 2G) as compared with NC groups. SOCE could be inhibited by SKF96365 in all groups (Fig 2E, 2F and 2G). The mean peak numbers of calcium responsive curves, which reflected the SOCE activity, were 3.2 ± 0.2, 5.2 ± 0.3, 3.1 ± 0.1 during 600 s in TSCCA, Hep2, and Tb3.1 cells, respectively. Peaks were nearly diminished when transfected with STIM1 siRNA and add SKF96365 (Fig 2H). For GV144-STIM1 Tb3.1 cell groups, the mean peak number was 5.8 ± 0.4, which was significantly more than the NC groups (*p*<0.05).

### 3.3 STIM1 and SOCE influence cell proliferation

**Fig 3:**
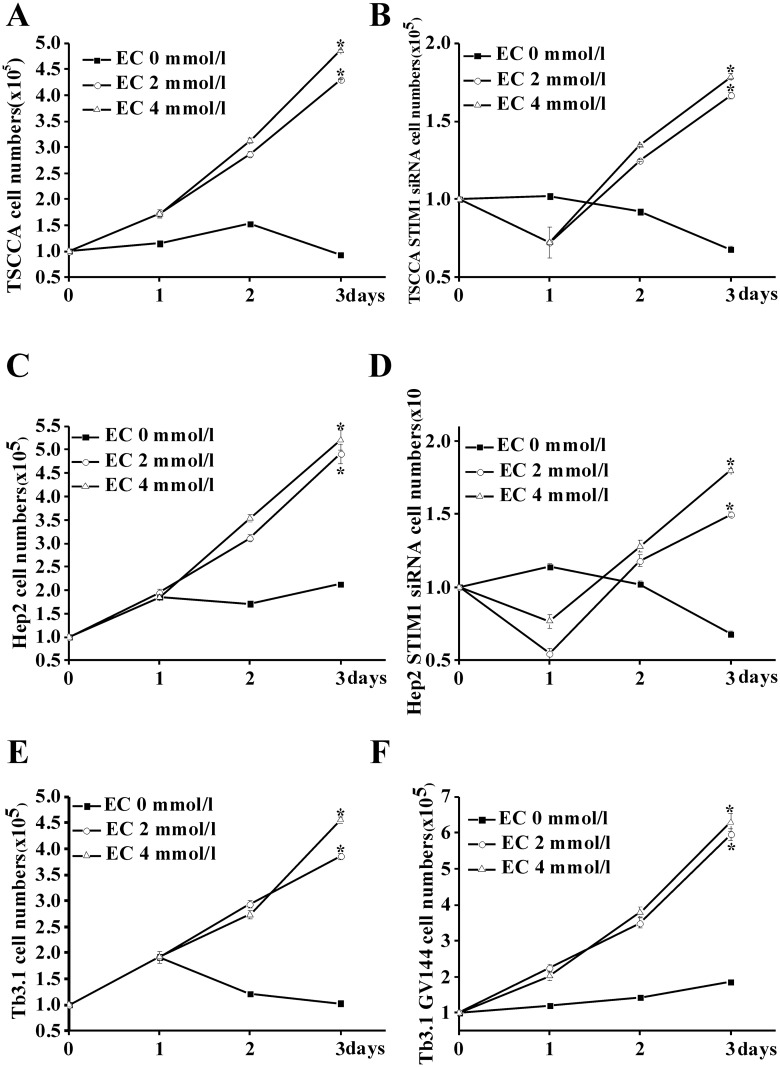
STIM1 expression influences the HNSCC cell proliferation. The extracellular calcium (EC) concentration were 0, 2 and 4 mmol/L respectively. (A)TSCCA cell proliferation during 3 days.* *p*<0.05 versus 0 mmol/L EC. (B)TSCCA STIM1-siRNA cell proliferation during 3 days. * *p*<0.05 versus 0 mmol/L EC. (C) Hep2 cell proliferation during 3 days.* *p*<0.05 versus 0 mmol/L EC. (D) Hep2 STIM1-siRNA proliferation during 3 days.* *p*<0.05 versus 0 mmol/L EC. (E) Tb3.1 cell proliferation during 3 days.* *p*<0.05 versus 0 mmol/L EC. (F) Tb3.1 STIM1-siRNA proliferation during 3 days.* *p* <0.05 versus 0 mmol/L EC.

As Fig 3A showed, extracellular calcium free culture medium would notably inhibite cell proliferation in TSCCA. This procession may partly induced by STIM1 due to downregulate STIM1 expression also inhibition cell proliferation (Fig 3B). For Hep2 cells (Fig 3C and 3D), it had similar results with TSCCA.

As Fig 3E showed, extracellular calcium free culture medium would notably inhibition cell proliferation in Tb3.1. Upregulated STIM1 expression by transfected with GV144 significantly promoted cell proliferation (Fig 3F).

Cell proliferation in all groups notably decreased as add SKF96365 in culture medium (S3 Fig).

### 3.4 STIM1 expression influence cell cycle

**Fig 4:**
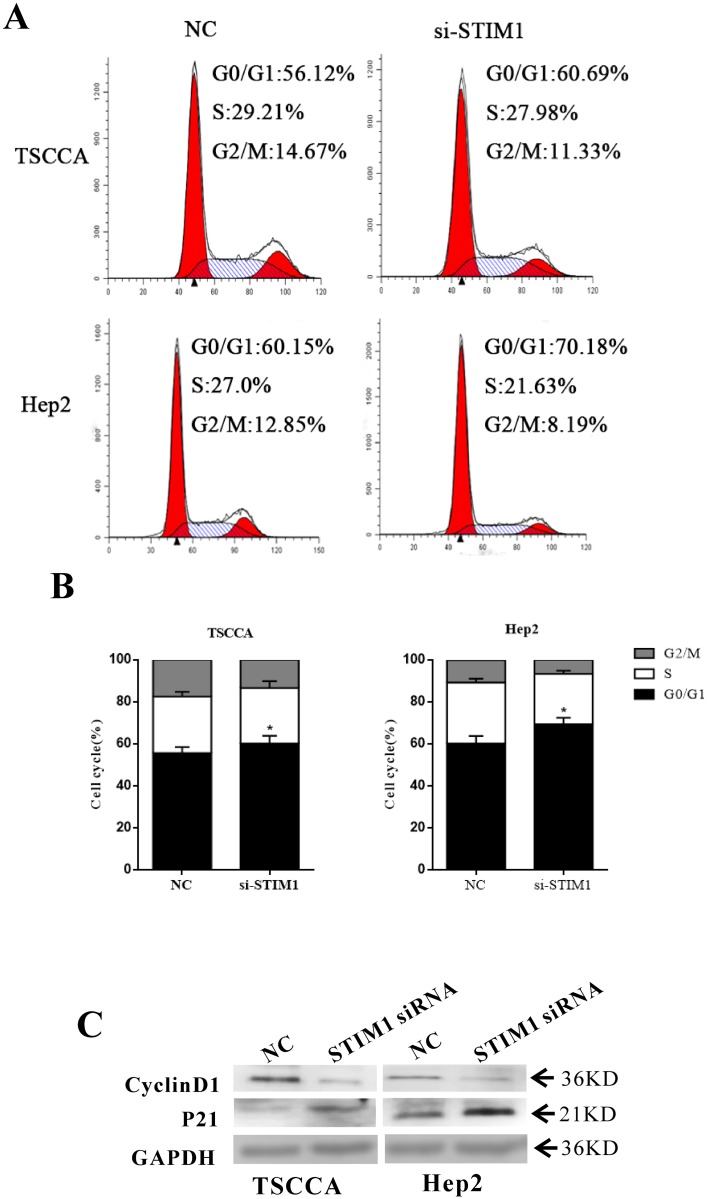
STIM1 expression influences the HNSCC cell phases. (A) FACS measurements showed that downregulated STIM1 expression arrested more cells in G0/G1 stage for TSCCA and Hep2. (B) Data analysis about Fig 4A. *p<0.05 (C) WB detection of CyclinD1 and P21. Results showed that CyclinD1 decreased in STIM-siRNA groups than in normal, whereas P21 increased in STIM1-siRNA groups for TSCCA and Hep2.

Cell cycle distribution analysis was detected by flow cytometry at 48 hours after transfection. The percentage of G0/G1 phase in the STIM1 siRNA groups were increased by 4.525% ± 0.045% and 10.12% ± 0.058% (*p*<0.05) at 48 hours in TSCCA and Hep2 respectively (Fig 4A) as compared with the NC group. Meanwhile, an increase of P21 protein and a decrease of cyclinD1 protein were noted in STIM1siRNA groups when compared with NC groups (Fig 4C).

### 3.5 ER stress induced by TG could be strengthen by downregulated STIM1 expression or attenuated by upregulated STIM1 expression in HNSCC cell lines

**Fig 5:**
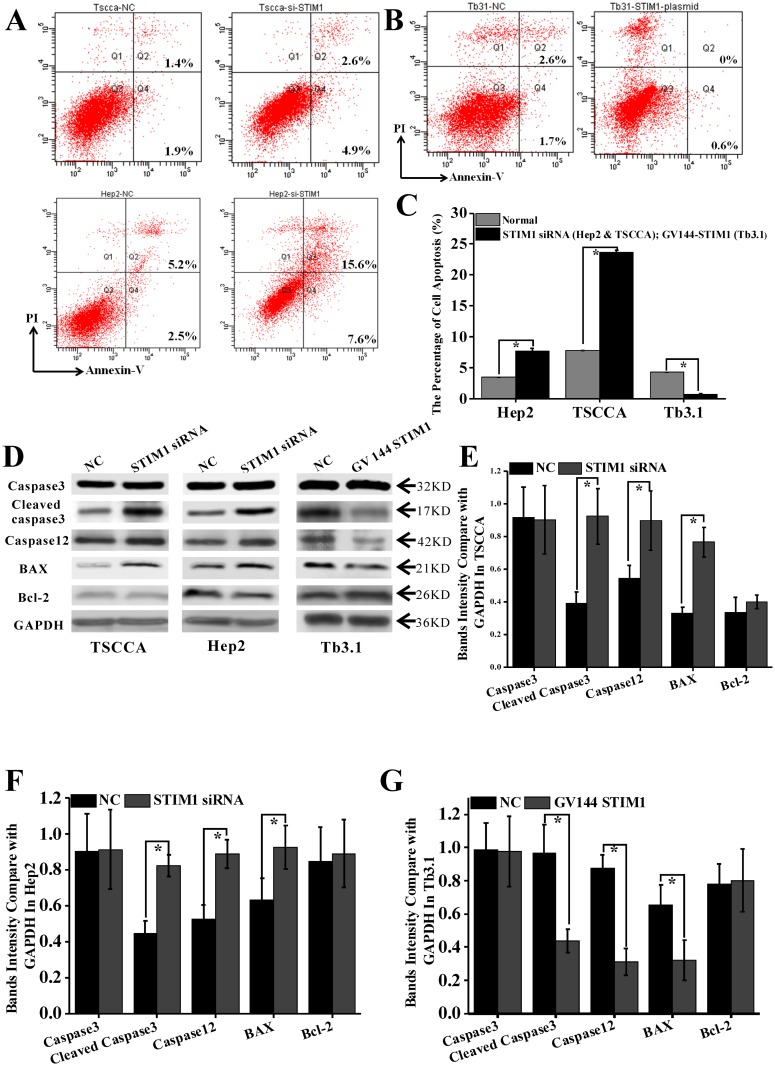
STIM1 expression closely related to HNSCC cell apoptosis and participated in TG-induced ER stress. (A) Transfected with STIM1-siRNA in TSCCA and Hep2 could significantly increase cell apoptosis compared with control groups by FACS detection. (B)Transfection with GV144 STIM1 in Tb3.1 significantly decrease cell apoptosis proportion compared with control groups by FACS detection. (C) Data analysis of A and B. * *P*< 0.05. (D) 10mmol/L TG was added in all groups to induce ER stress. BAX, cleaved caspase3, and caspase12, Bcl2 were detected using WB detection. (E) Cleaved caspase3, caspase12, and BAX were notably increased in STIM1-siRNA groups than in normal for TSCCA. Caspase3 and Bcl2 expression had no difference among the two groups. (F) Cleaved caspase3, caspase12, and BAX were notably increased in STIM1-siRNA groups than normal for Hep2. Caspase3 and Bcl2 expression had no difference among the two groups. (G) Cleaved caspase3, caspase12, and BAX were notably decreased in GV144-STIM1 groups than in normal for Tb3.1. Caspase3 and Bcl2 expression had no difference among the two groups.

As shown in FACS, the proportion of cell apoptosis in TSCCA and Hep2 cells transfected with STIM1 siRNA groups were significantly increased by 4.29%±0.44% and 15.1% ± 1.06%, respectively, compared with NC groups (*p*<0.05; Fig 5A). For Tb3.1 cells transfected with GV144-STIM1, the apoptosis proportion was notably decreased compared with NC groups (0.5% ± 0.1% vs. 4.32% ± 0.42%, *P*<0.001; Fig 5B).

Calcium-related cell death may partly result from ER stress, because calcium homeostasis in ER may induce ER stress. Some factors, such as palmitate, can induce ER calcium depletion and apoptosis in mouse podocytes subsequent to mitochondrial oxidative stress [27]. Caspase 12 and cleaved caspase3 were sequential be activated in ER stress, then cells developed into death. The results showed that the expression levels of caspase12, cleaved caspase3, and BAX in STIM1 siRNA groups were notably higher than those in NC groups for TSCCA and Hep2 cells under 10 μm/L TG stimulation (Fig 5E and 5F). On the contrary, those indexes in Tb3.1 cells transfected with GV144-STIM1 groups were lower than those in the NC groups (Fig 5D and 5G; *P<*0.05). Bcl2 expression levels in the transfected groups had no statistical difference with that of the NC (Fig 5D, 5E, 5F and 5G).

### 3.6 Effects of STIM1 silencing on tumor growth in vivo

**Fig 6:**
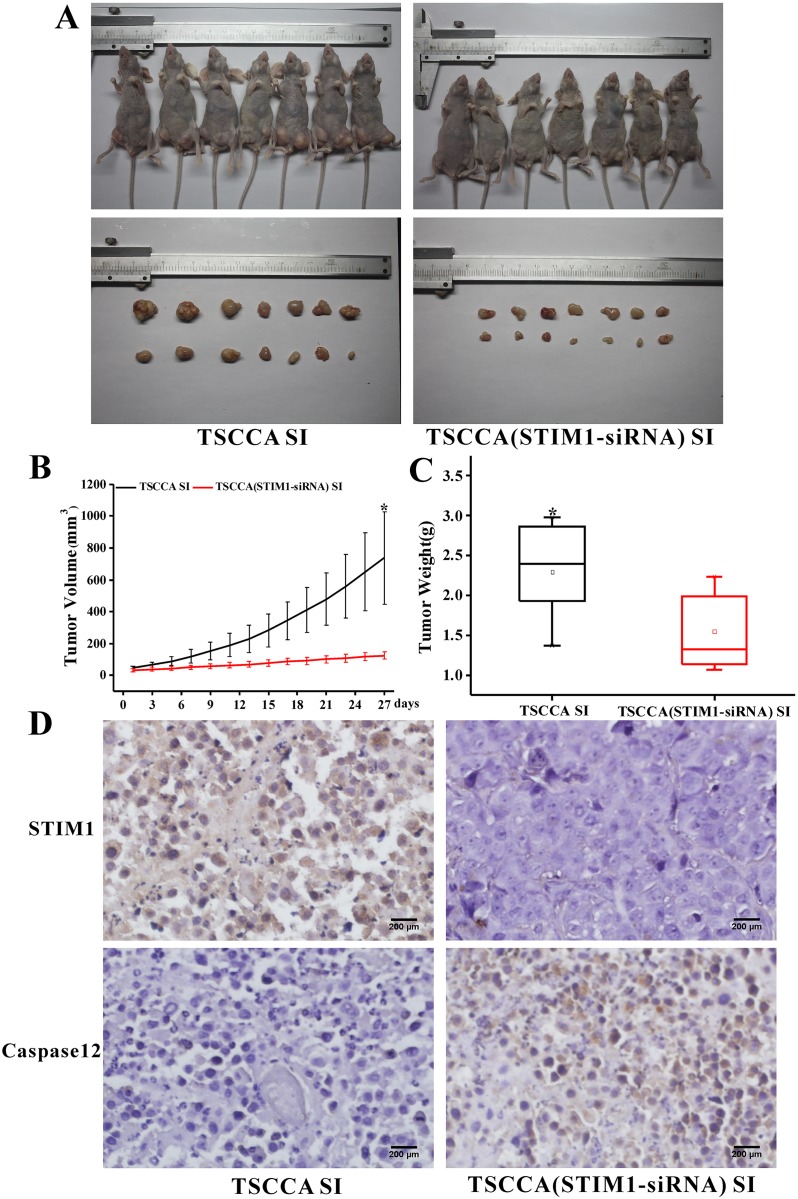
Downregulated STIM1 expression *in vivo* TSCCA xenograft could inhibit tumor growth. (A) Establish mouse oral cancer model by subcutaneous injection TSCCA or TSCCA with STIM1-siRNA. Images of TSCCA SI and TSCCA (STIM1-siRNA) SI-treated xenograft tumors. (B) Growth curves of xenograft tumors. *P< 0.05 (C) Tumor weight of xenograft tumors *P<0.05. (D) IHC staining of STIM1 and caspase12 in two groups. Yellow staining for STIM1 and caspase12.

Tumorigenicity experiment was performed in nude mice using TSCCA cell xenograft model to confirm the function of STIM1 *in vivo*. Seven mice were collected for TSCCA STIM1-siRNA cells treated group (experiment group), and another seven mice were treated with TSCCA as control group. The mice were monitored and treated once every 3 days for 4 weeks. Compared with the control group, the experiment group showed significantly decreased tumor volume and weight (*p*<0.05; Fig 6A–6D).

STIM1 expression in IHC staining was elevated in TSCCA SI groups, while caspase 12 expression was notably high in TSCCA (STIM1-siRNA) SI groups. The expression of caspase3, caspase12, BAX, and P21 were upregulated, whereas Bcl2 and cyclinD1 were downregulated in the TSCCA SI group compared with that in the TSCCA STIM1-siRNA groups, according to the IHC staining results (S2 Fig). Those results were coincident with in vivo results

## Discussion

Local recurrence and distant metastasis were two major causes of HNSCC treatment failure. Despite many studies on related molecular mechanism, knowledge on its relationship with intracellular Ca^2+^ signals and related channels is limited. STIM1, which was an important channel for extracellular calcium influx, showed higher expression in many cancer tissues, such as breast cancer[28], colorectal cancer[20], cervical cancer [18]and hepatocarcinoma[29]. STIM1 overexpression usually promote cancer procession and metastasis. However, knowledge on the role of Ca^2+^ signal and STIM1 in HNSCC development is limited until recently. Our clinical studies showed that approximately 89% of HNSCC display STIM1 overexpression in tumor tissues and had close linear relationship with tumor sizes but not with neck lymph node metastasis. Thus, this study focuses more on the role of STIM1 and SOCE in cell proliferation and apoptosis for HNSCC *in vitro* and *in vivo* and highlights the novel role of Ca^2+^ store-sensor STIM1 in HNSCC.

STIM1 and SOCE participate in cell cycle and proliferation in HNSCC cell lines. Downregulated STIM1 expression by transfected with STIM1-siRNA would notably decrease cyclinD1 expression level but increase P21 expression level for TSCCA and Hep2 cells. The percentage of cells that arrest in G0/G1 stages significantly increased, whereas cell proliferation was inhibited in this condition. By contrast, upregulated STIM1 expression by GV144-STIM1 would significantly promote cell proliferation. Overexpression of cyclinD1 was closely related to hepatocellular carcinoma[30], esophageal squamous cell carcinoma[31] and laryngeal carcinoma[32] procession and could promote tumor cell proliferation and arrest cell in G1/S phase, P21 inhibition tumor cell growth. In addition to the influence on cell cycle, calcium free, blocking STIM1 expression or add SKF96365 could greatly inhibit cell proliferation. Therefore, STIM1 and calcium signal could be a target pathway for HNSCC treatment.

STIM1 may be selected as an anti-apoptosis biomarker for HNSCC cell lines. TG usually used to induce ER stress in many types of cells, such as mouse 3T3-L1 adipocytes [33] and COS7 cells [34]. In the present study, TG also induced ER stress in TSCCA and Hep2 cells as its marker (caspase12) elevated. Furthermore, downregulated STIM1 expression by STIM1-siRNA would significantly accelerate this process. By contrast, Tb3.1, which had low STIM1 expression, showed relative stronger ER stress response under TG stimulation. However, upregulated STIM1 expression by GV144-STIM1 in Tb3.1 notably attenuated ER stress response as the expression of cleaved caspase3 and caspase12 were significantly decreased (Fig 5D, 5E, 5F and 5G). These results demonstrated that high intracellular calcium concentration, which mainly maintained by STIM1 overexpression, can partly resist ER stress. Conversely, decreased intracellular calcium concentration by downregulating STIM expression may accelerate ER stress-related cell death.

Animal experiment was conducted to further verify above results. Tumor size and weight in nude mice was substantially inhibited in STIM1-siRNA group compared with the control group *in vivo*. Meanwhile, the expression change of cyclinD1, P21, and caspase12 in IHC staining were coincided with *in vitro* results, which further demonstrated that STIM1 and SOCE participate in HNSCC growth and anti-apoptosis process.

In conclusion, this study demonstrated that overexpression of STIM1 in HNSCC tissue has significant influence on tumor cell growth and apoptosis *in vitro* and *in vivo*. STIM1-mediated signaling is an attractive target for therapeutic intervention. Due to the present clinical data showed no STIM1 expression difference between neck lymph node metastasis positive and negative groups, thus we didn’t focus on its role in metastasis, but this may also be an important process that requires study in the future.

## Supporting information

S1 Fig Calcium response under TG stimulation in TSCCA cells. (TIFF)

S2 Fig IHC staining for BAX, Bcl-2, Caspase3, Cyclin D1 and P21 in mouse tumor tissue. (TIFF)

S3 Fig Cell proliferation curves for TSCCA, Hep2 and Tb3.1 as add SKF96365 under 2 or 4 mmol/l extracellular calcium concentration. (TIF)

S1 Text Origin documents. (DOCX)

S2 Text Animal origin. (XLSX)

S3 Text WB. (DOCX)

S4 Text IHC. (DOCX)
