# Supplementary figures and images for: Blocking of stromal interaction molecule 1 expression influence cell proliferation and promote cell apoptosis in vitro and inhibit tumor growth in vivo in head and neck squamous cell carcinoma
Source: PLoS One. 2017 May 11;12(5):e0177484. doi: 10.1371/journal.pone.0177484 (PMC5426681; doi:10.1371/journal.pone.0177484)

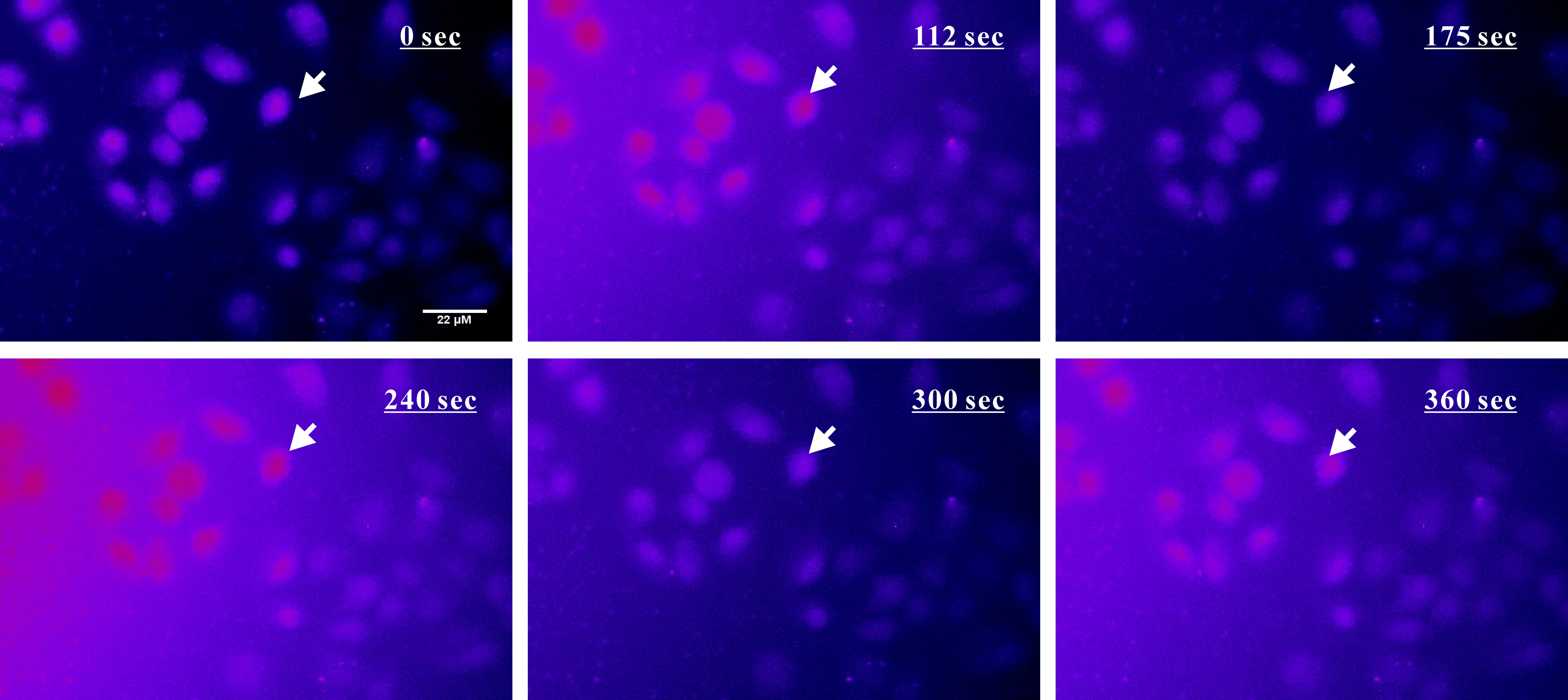

Supplement: S1 Fig — (TIFF) [file pone.0177484.s001.tiff]

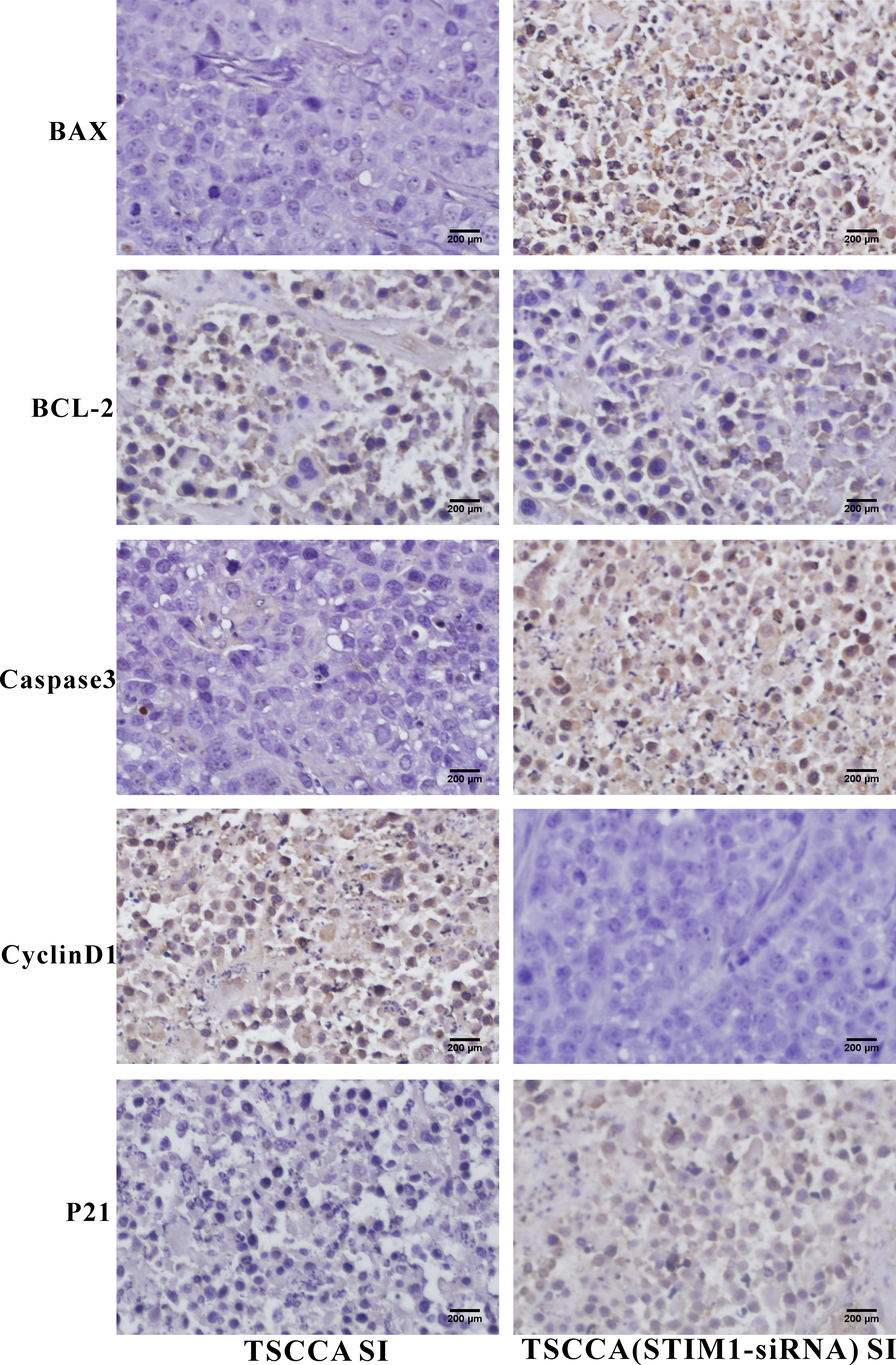

Supplement: S2 Fig — (TIFF) [file pone.0177484.s002.tiff]

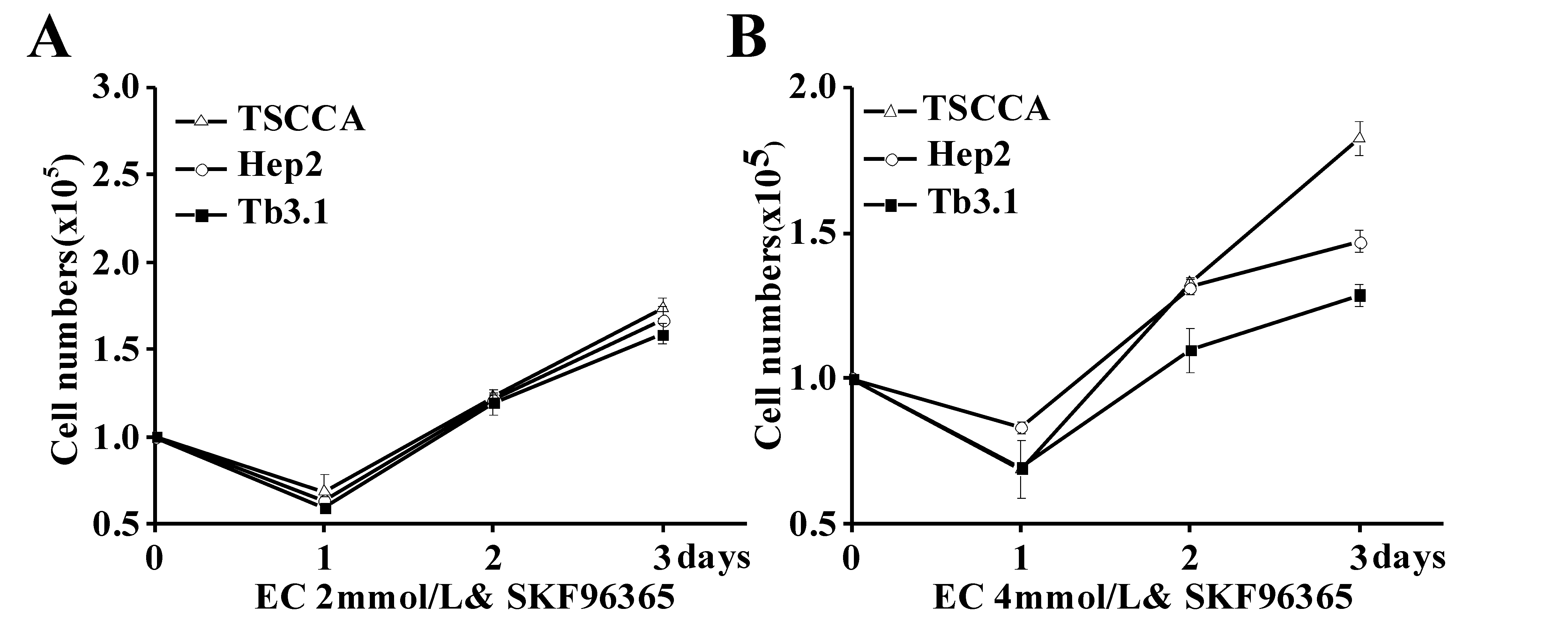

Supplement: S3 Fig — (TIF) [file pone.0177484.s003.tif]

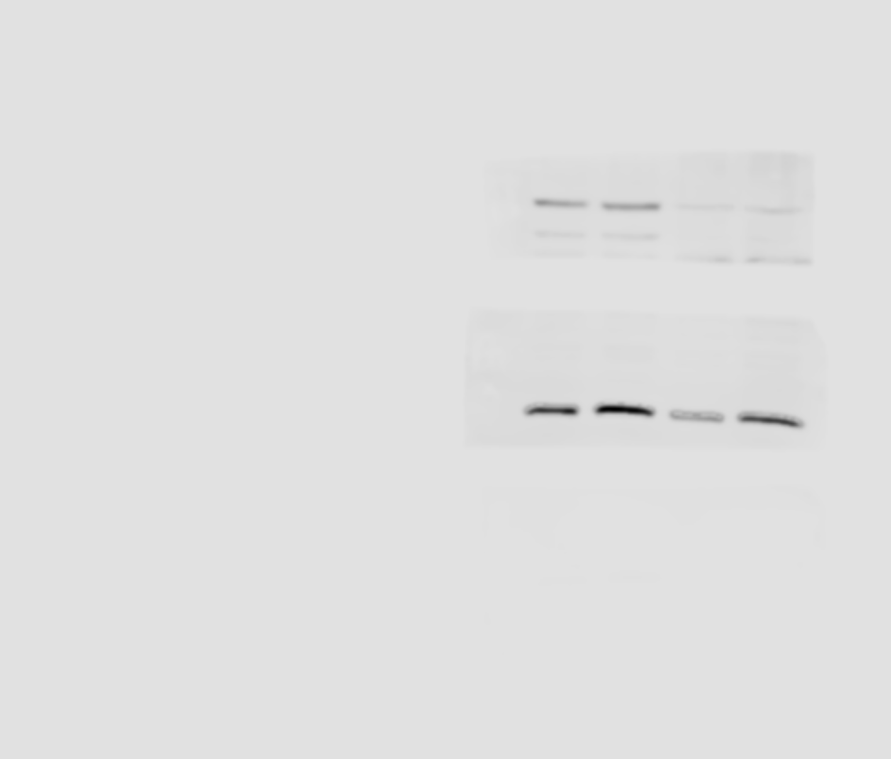


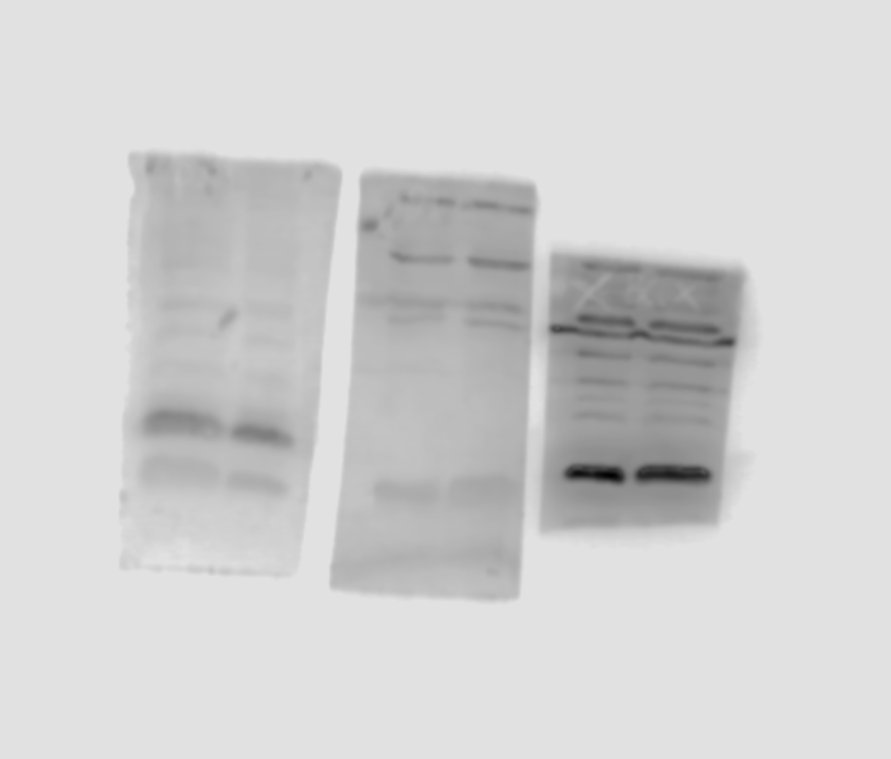


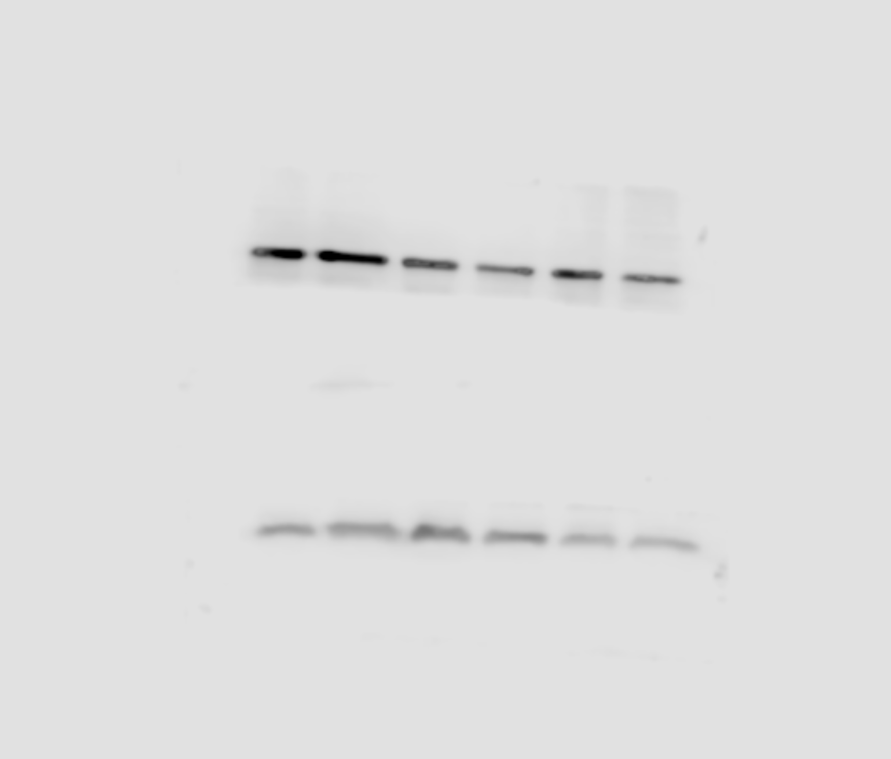


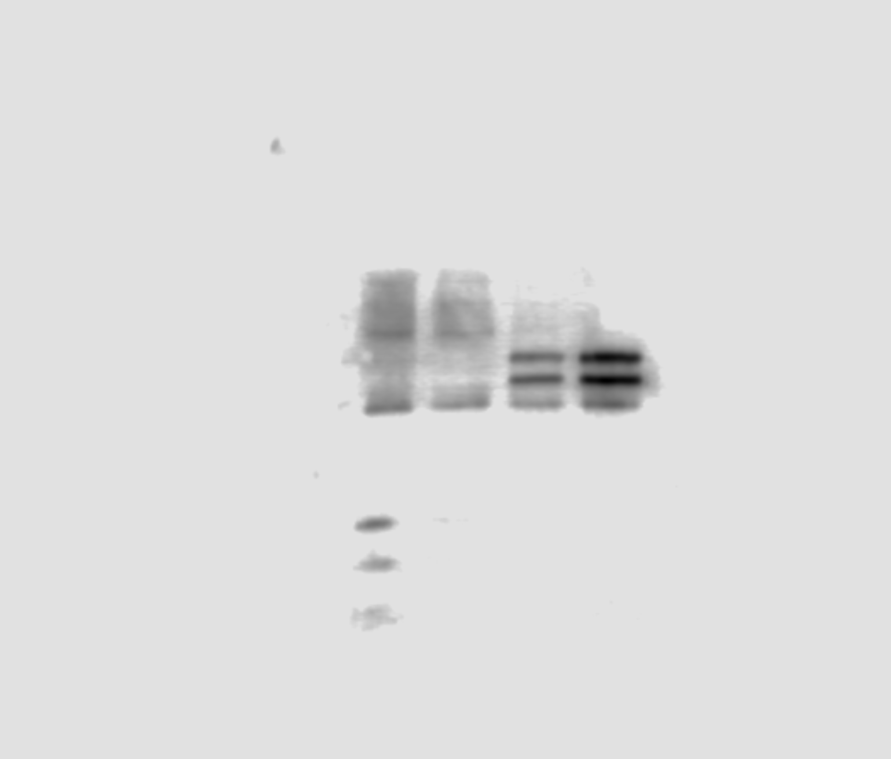


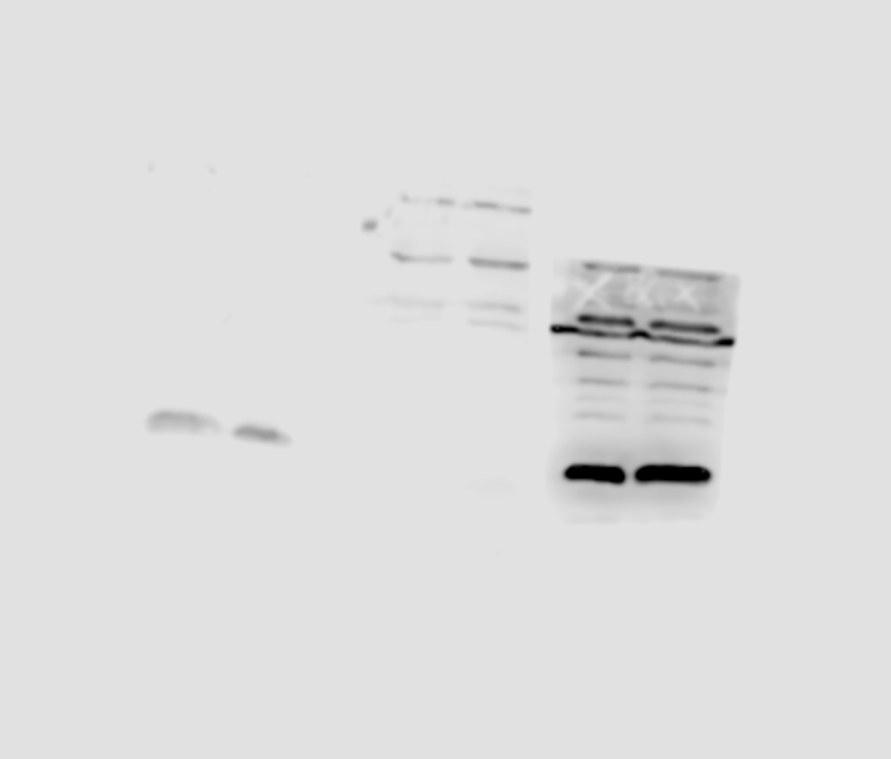


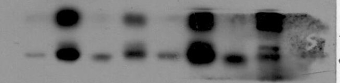


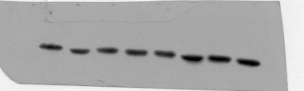


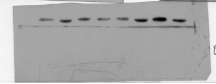


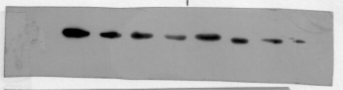

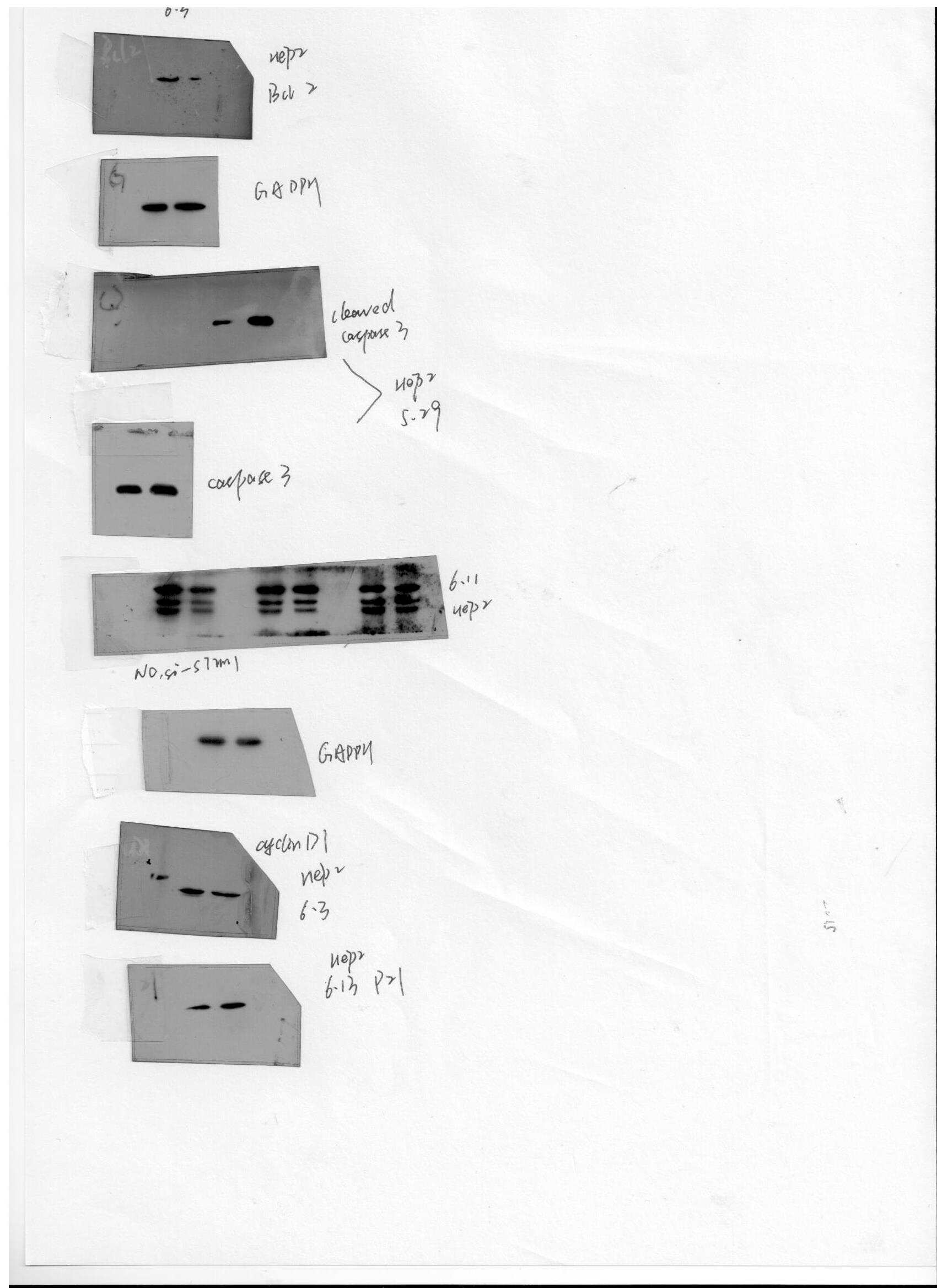


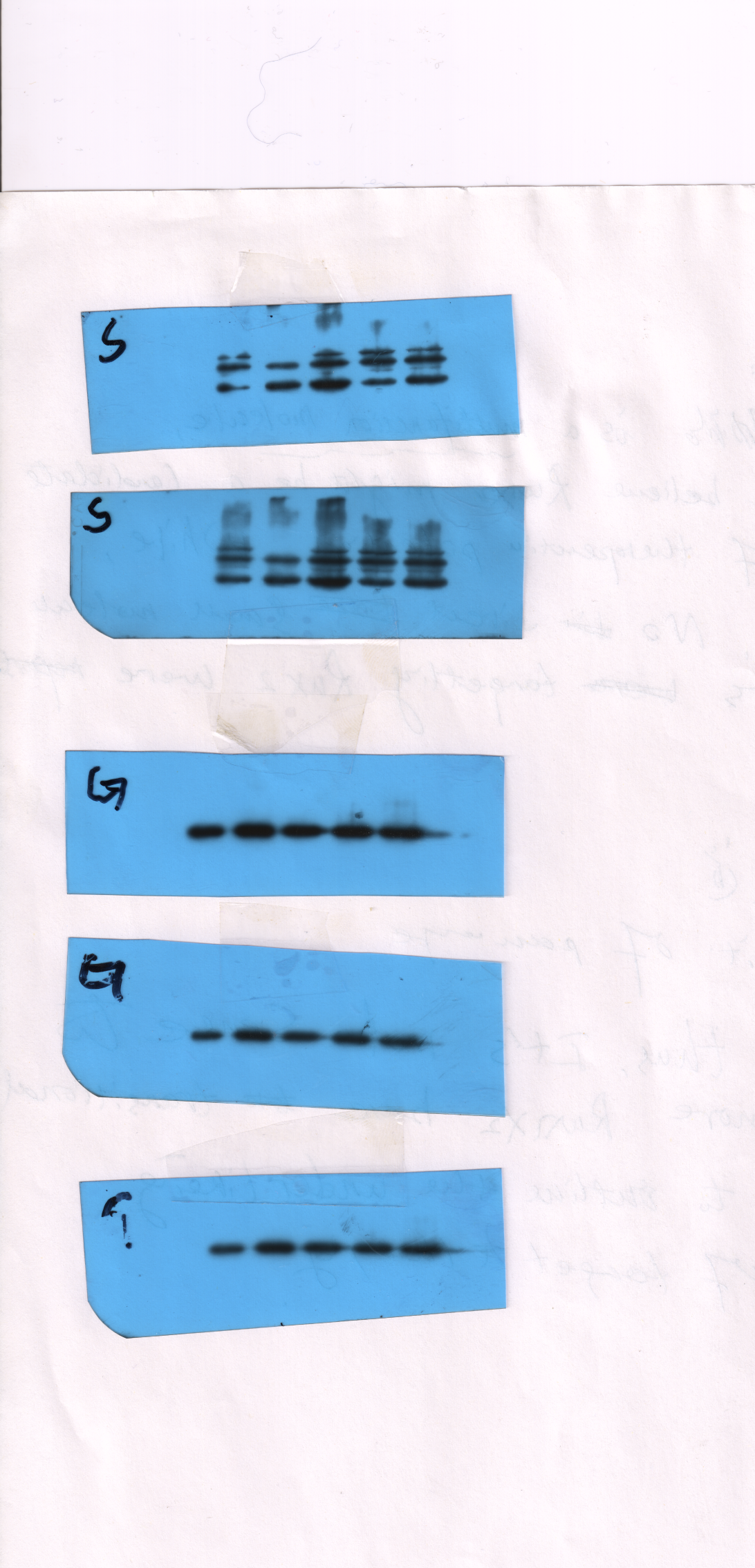


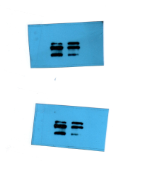


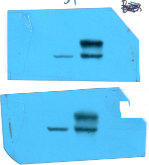


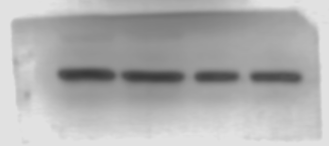


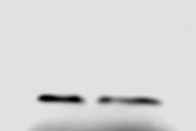


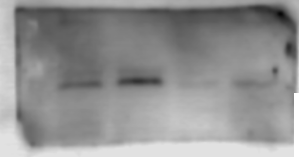


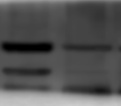


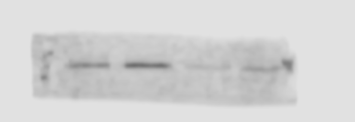


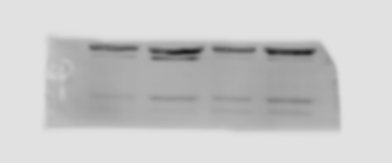


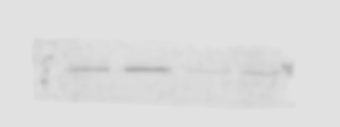


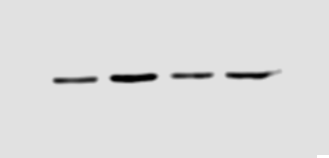


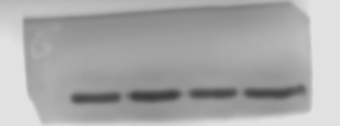


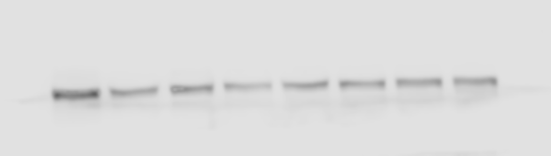

Supplement: S3 Text — (DOCX) [file pone.0177484.s006.docx]

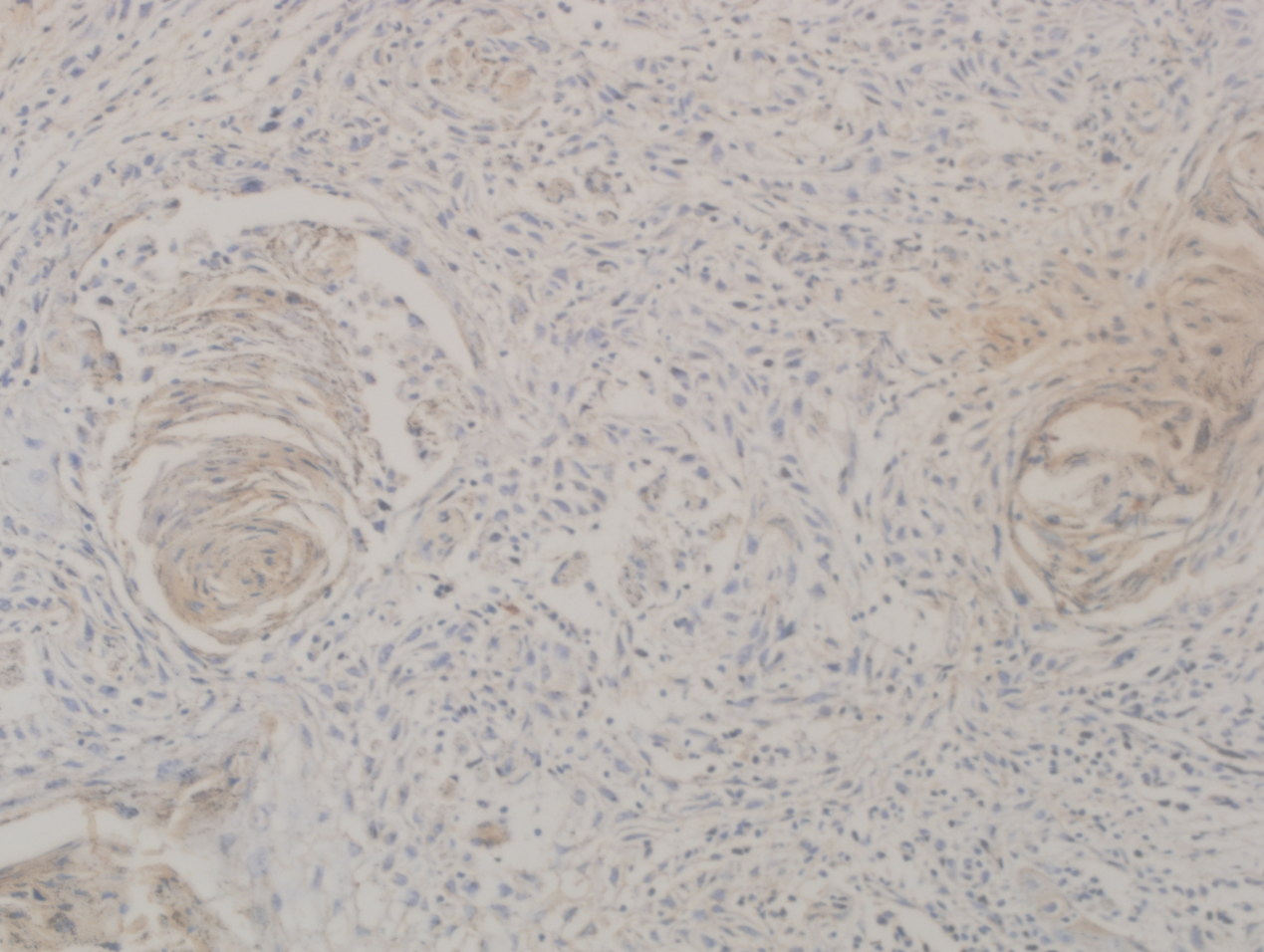


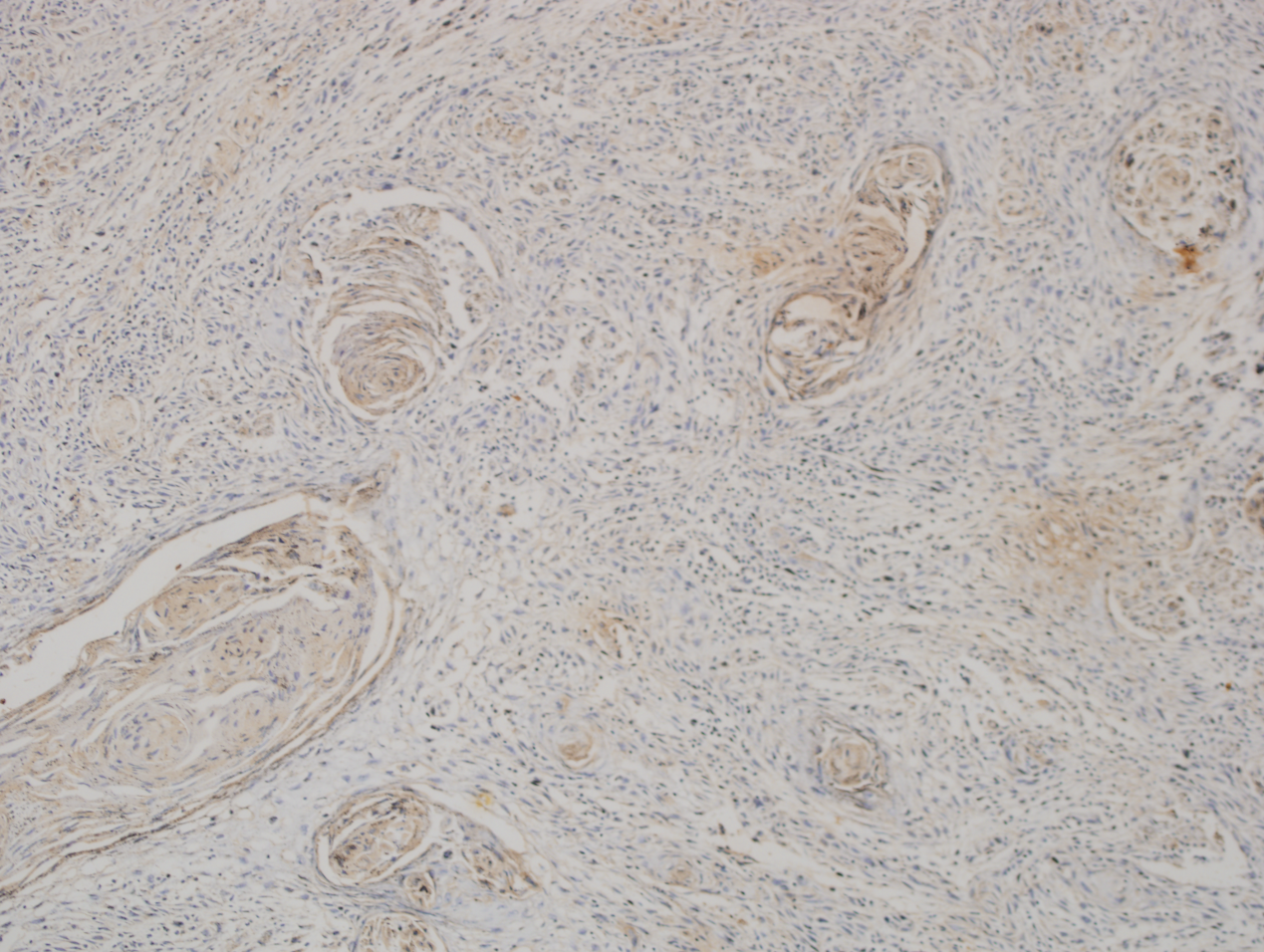


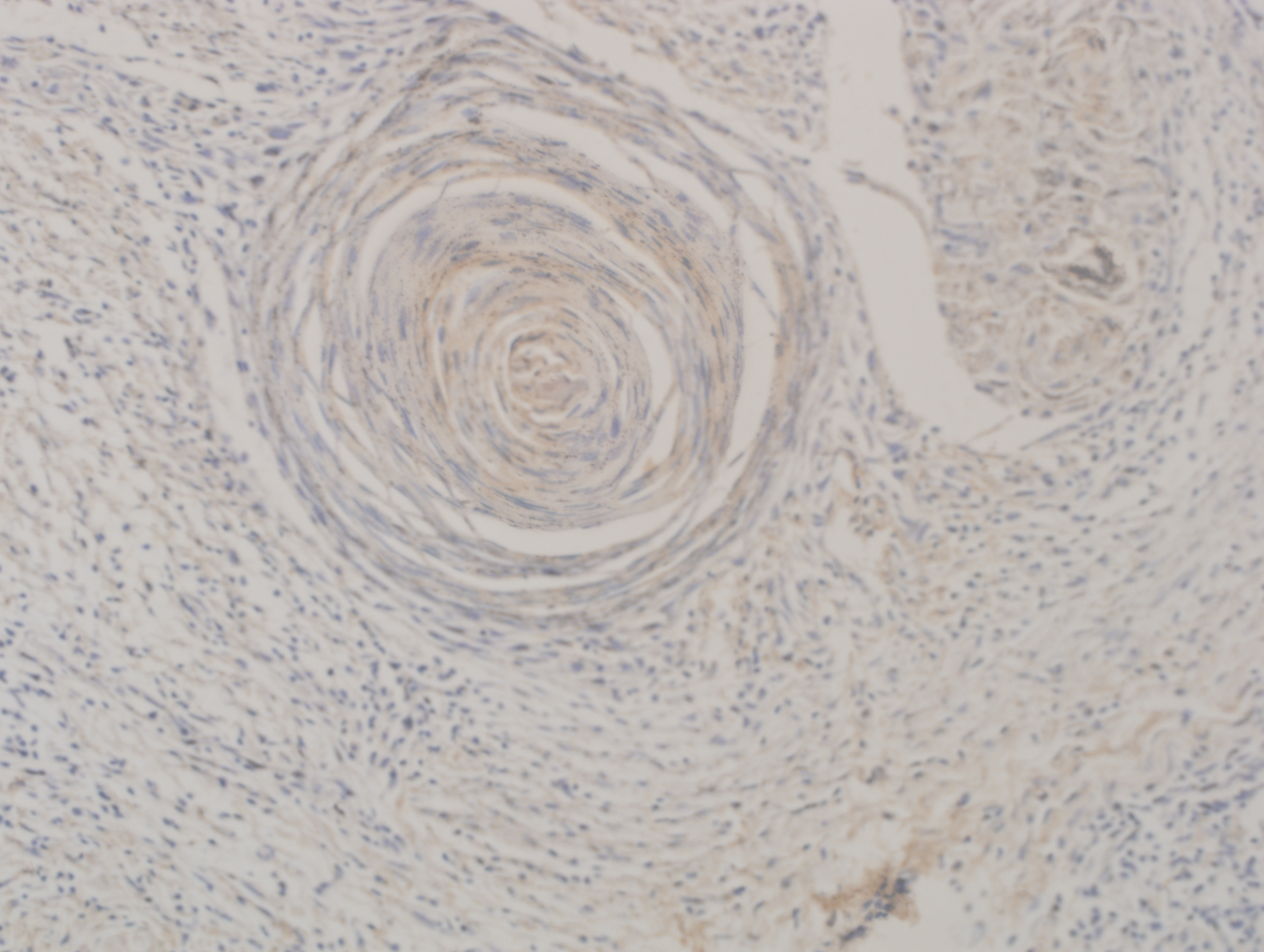


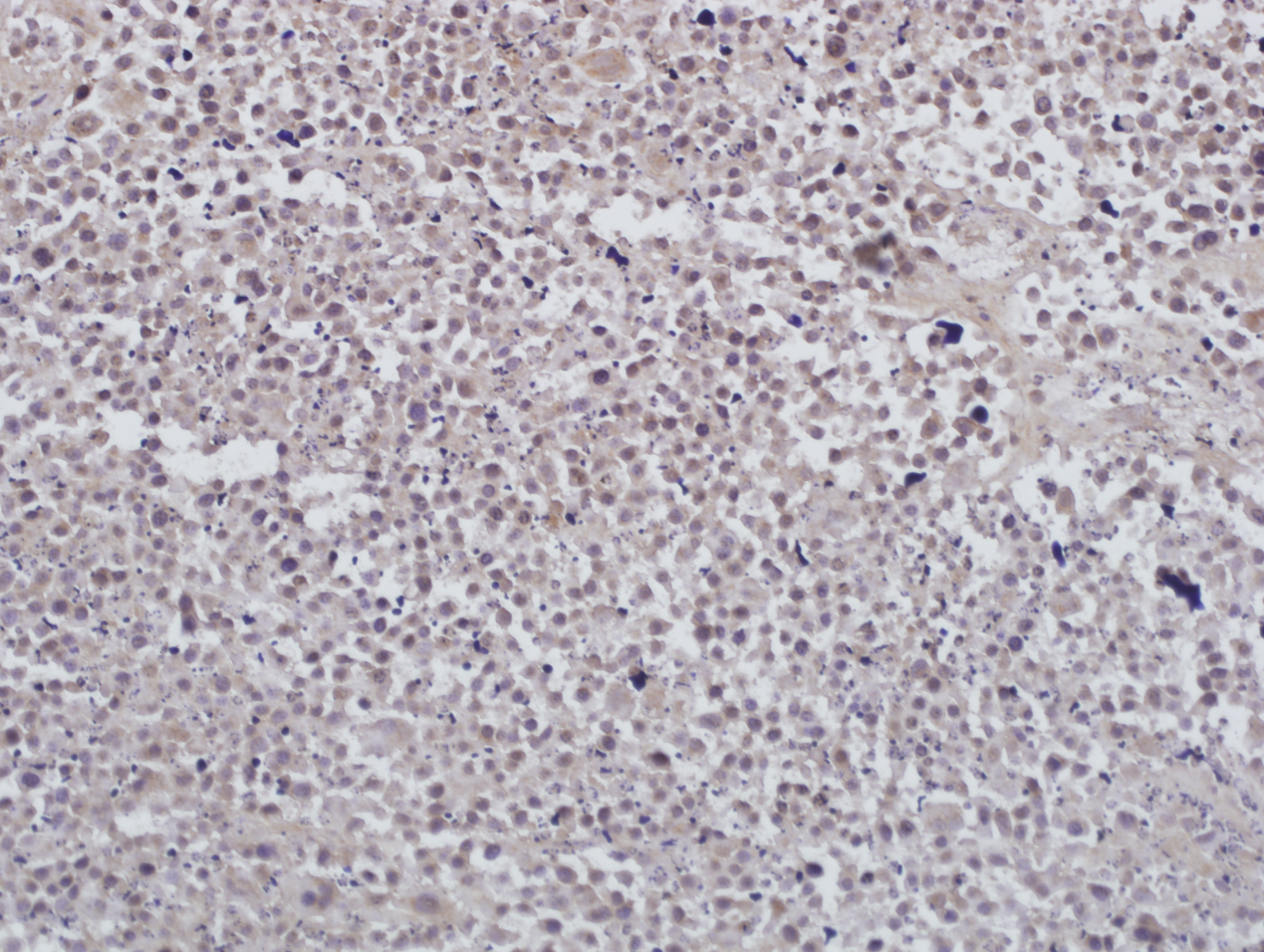


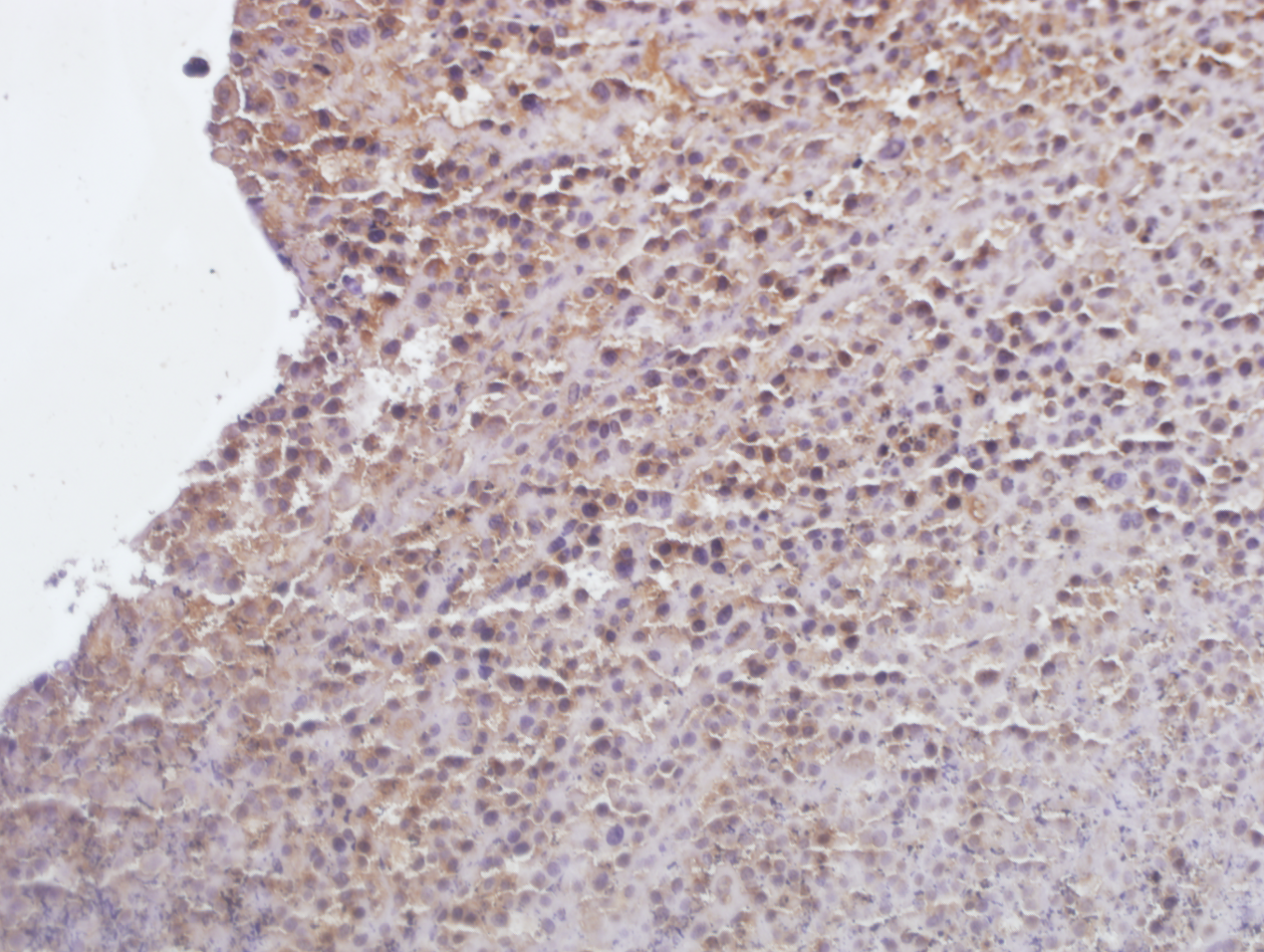


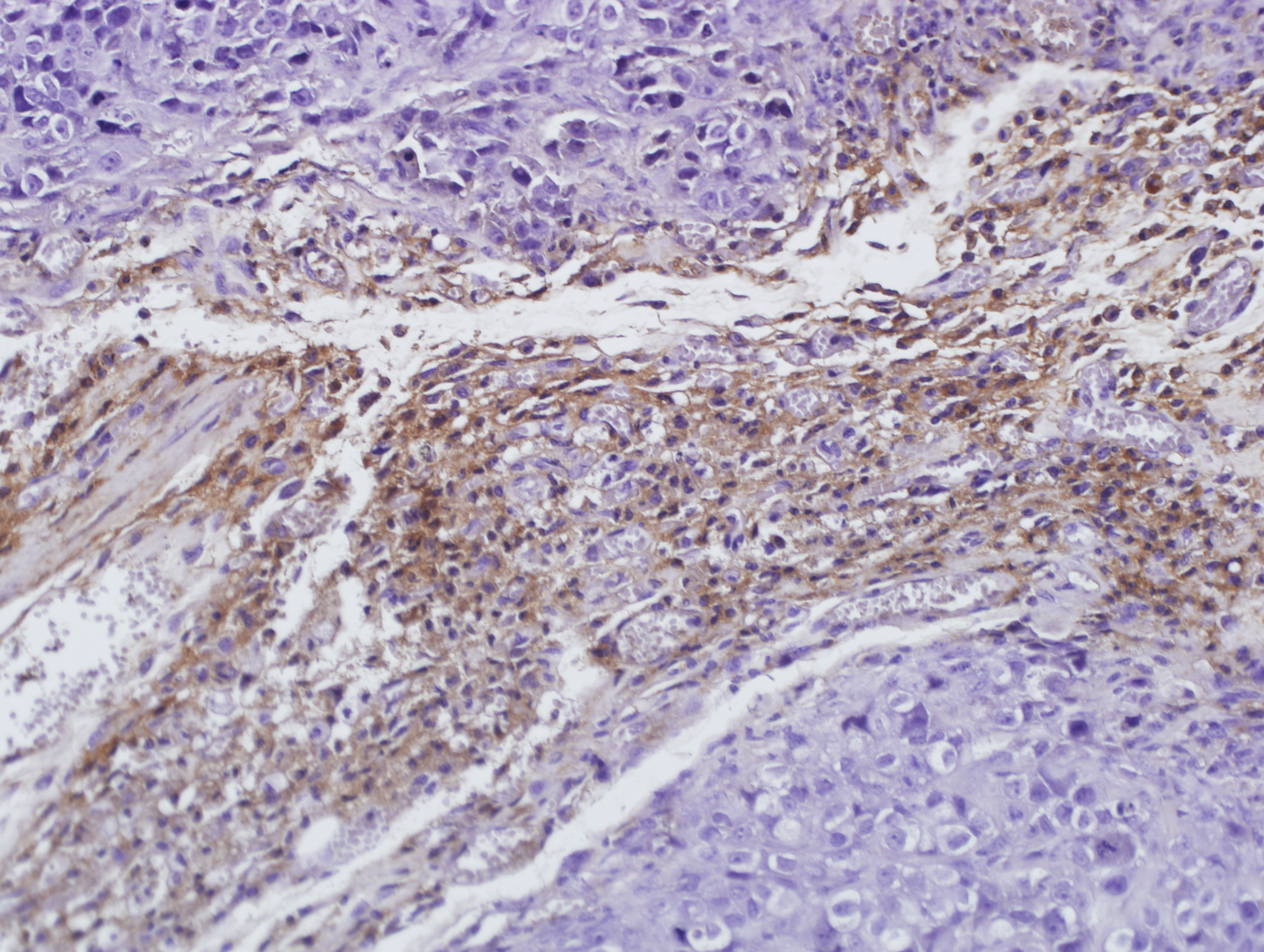


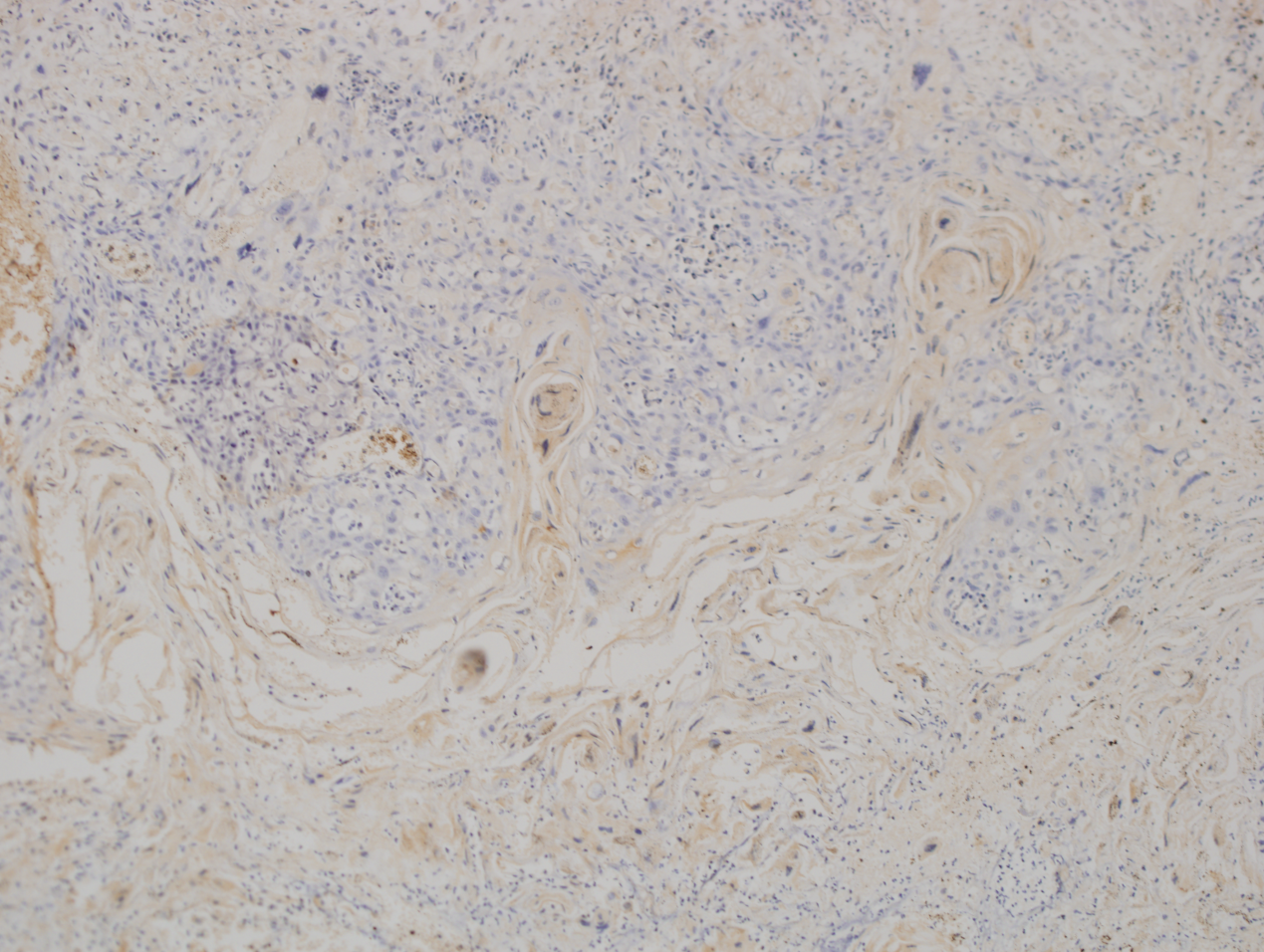


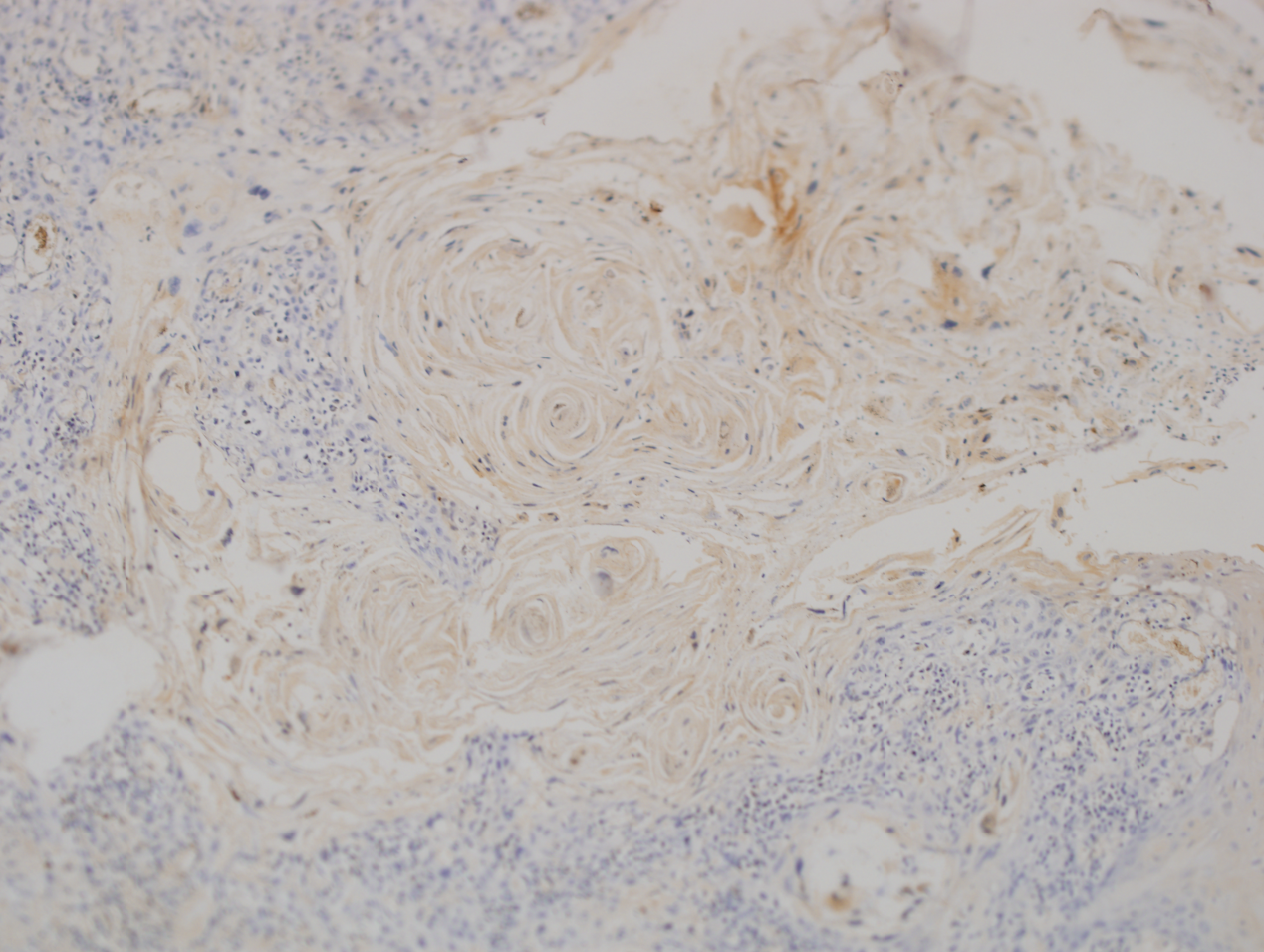


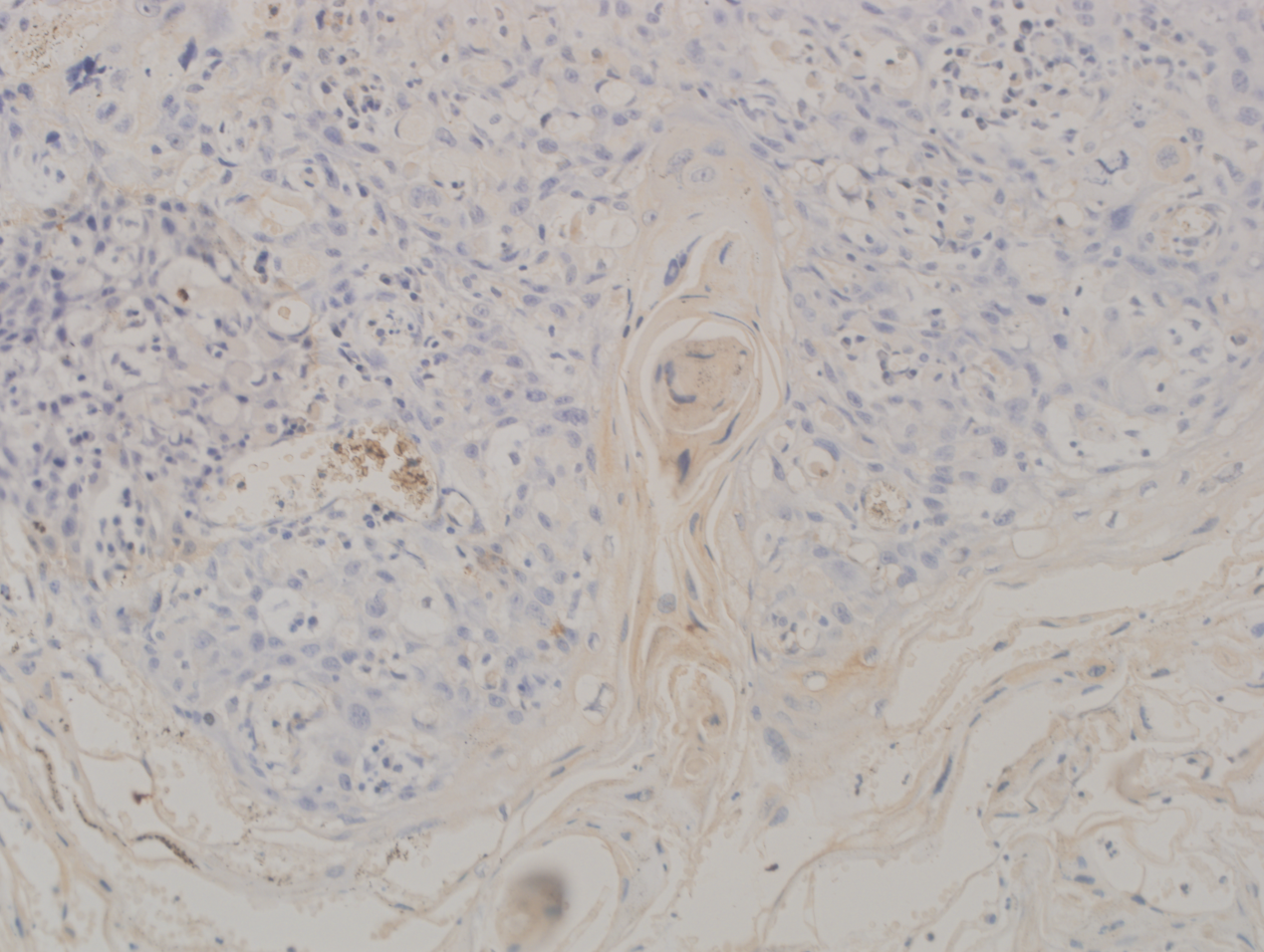

Supplement: S4 Text — (DOCX) [file pone.0177484.s007.docx]
