# Supplementary material for: Blocking of stromal interaction molecule 1 expression influence cell proliferation and promote cell apoptosis in vitro and inhibit tumor growth in vivo in head and neck squamous cell carcinoma
Source: PLoS One. 2017 May 11;12(5):e0177484. doi: 10.1371/journal.pone.0177484 (PMC5426681; doi:10.1371/journal.pone.0177484)
Supplement: S1 Text — (DOCX) [file pone.0177484.s004.docx]

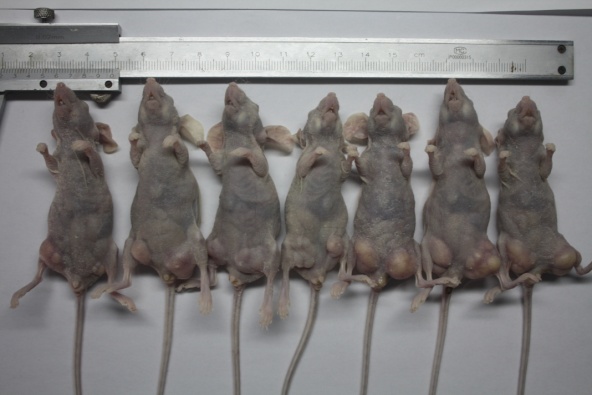

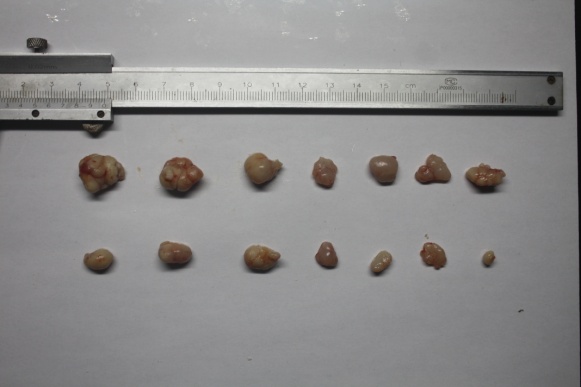


Control


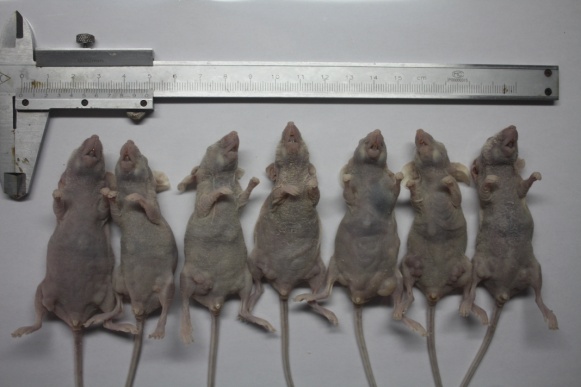

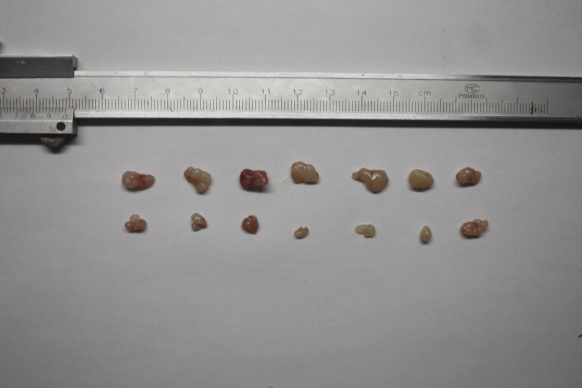


Si-STIM1


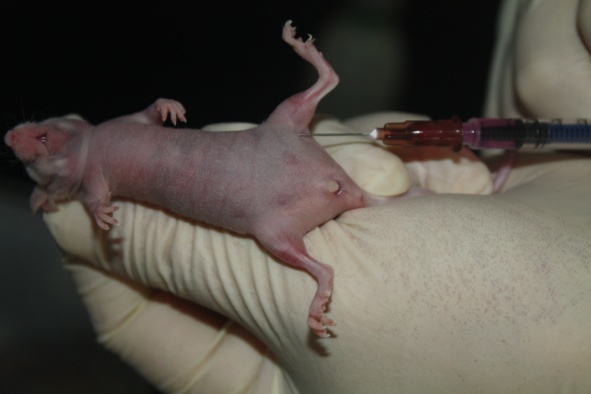

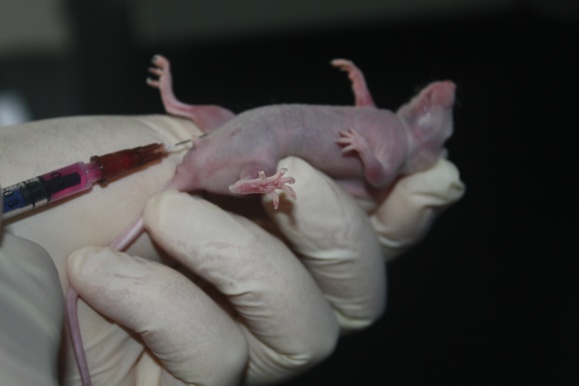


IHC (from human)


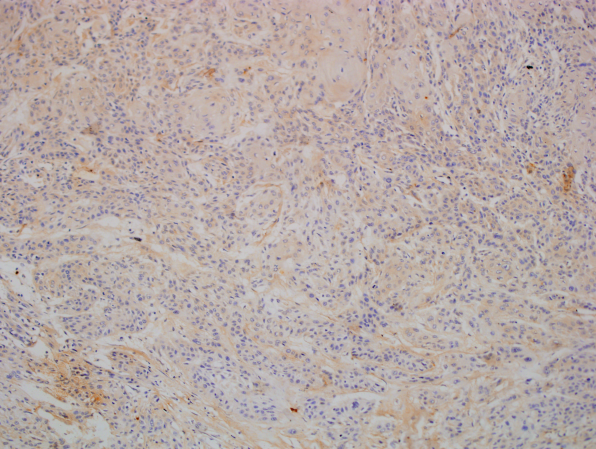

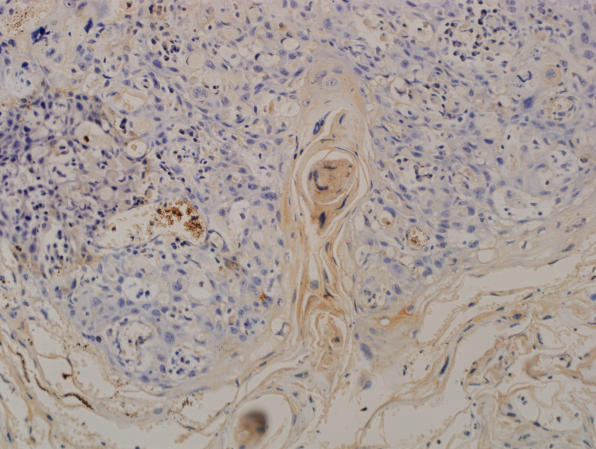


CA

CA


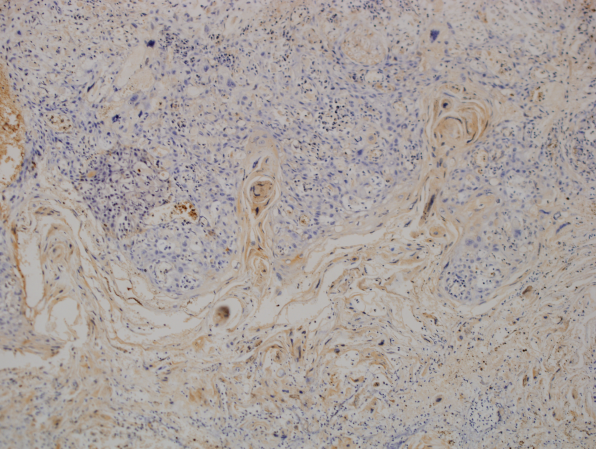

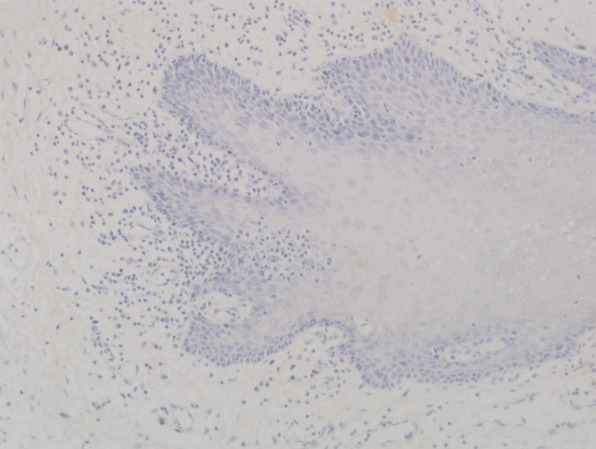


CAP

CA


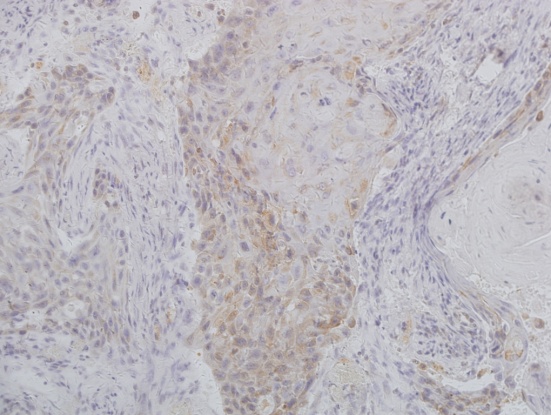

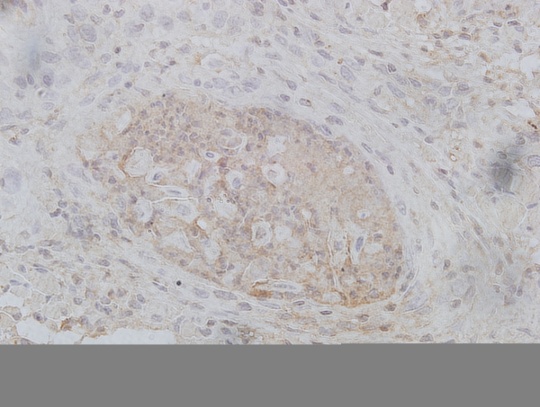


CA

CA


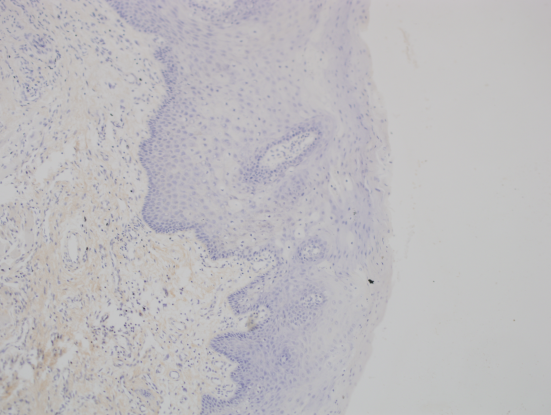

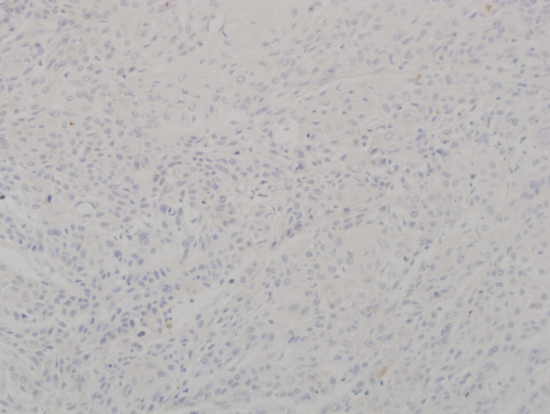


CAP

CAP


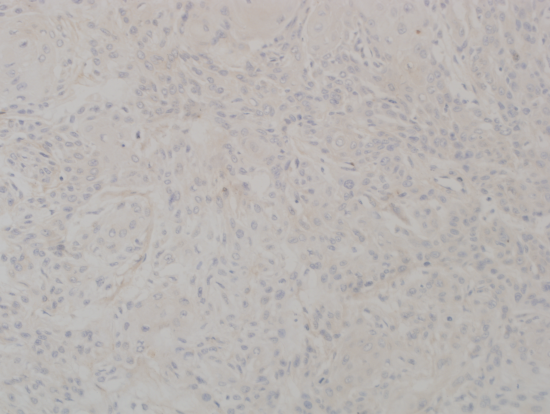

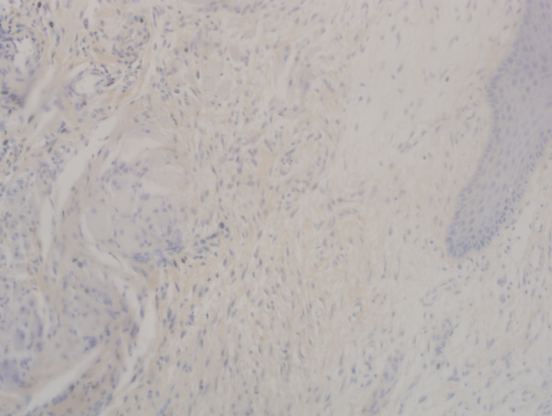


CAP

CAP

IHC (from annimal)


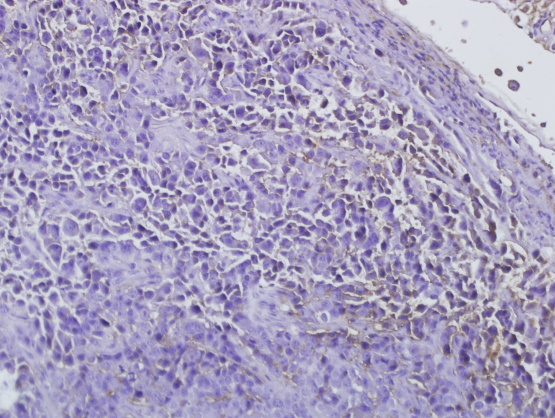

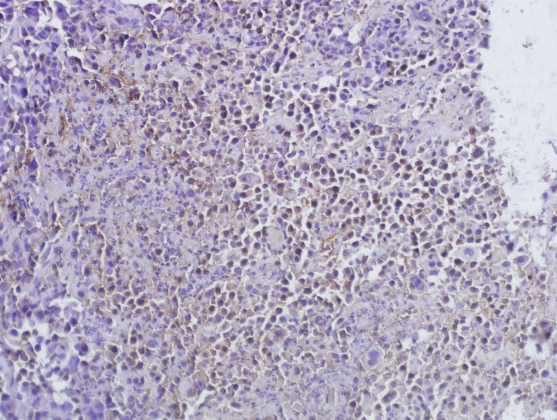


BAX NC

BAX si-STIM1


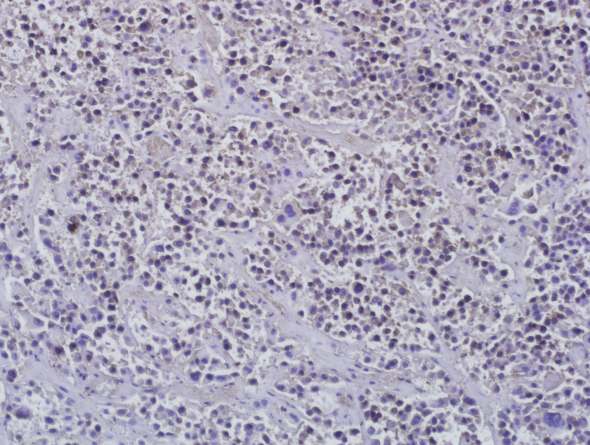

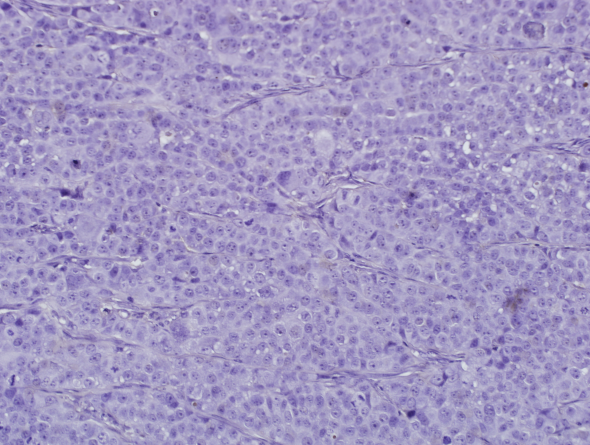


Bcl2 si-STIM1

Bcl2 NC


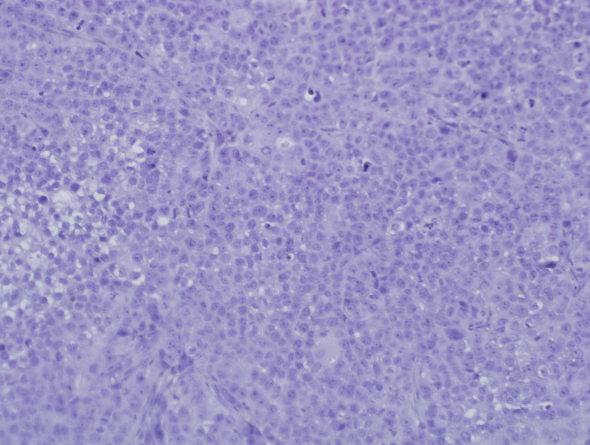

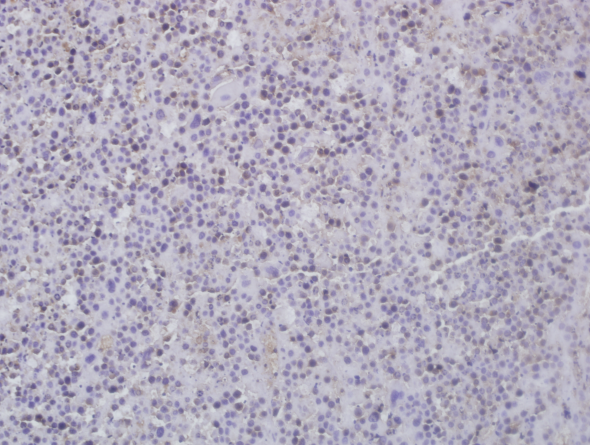


caspase3 si-STIM1

caspase3 NC


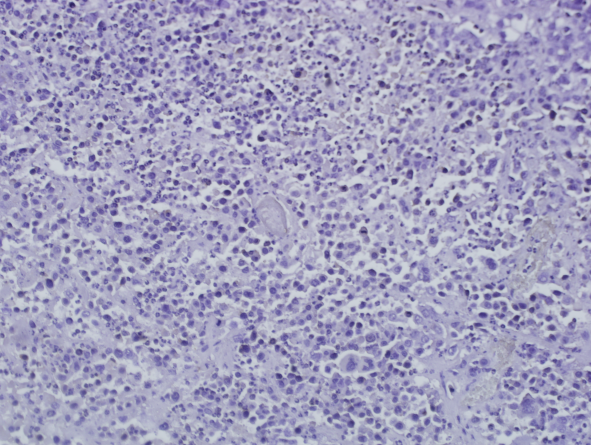

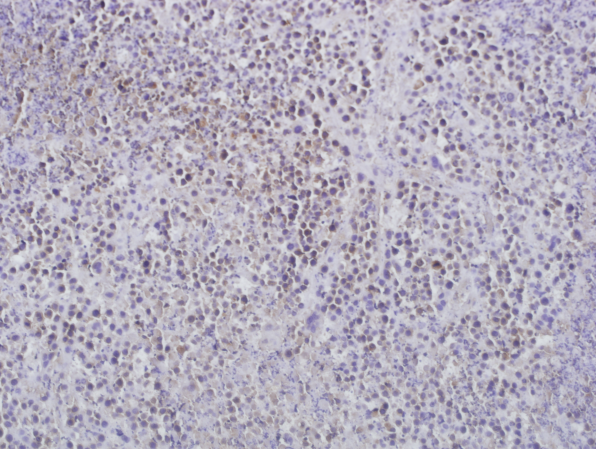


caspase12 si-STIM1

caspase12 NC


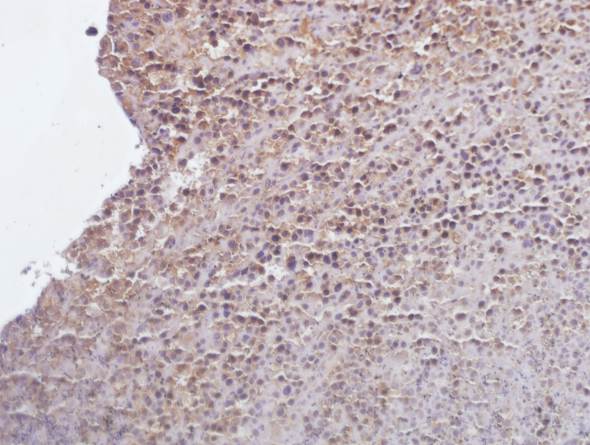

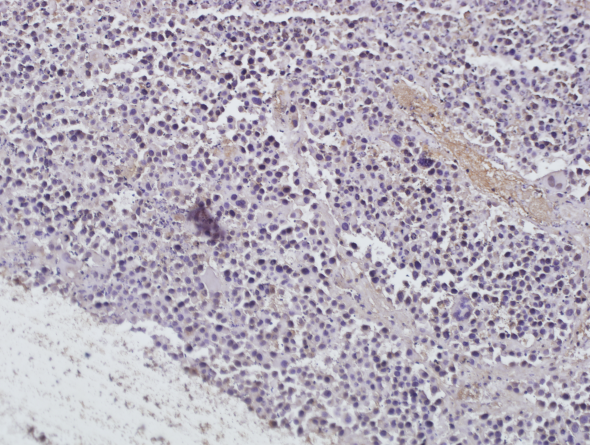


cyclinD1 si-STIM1

cyclinD1 NC


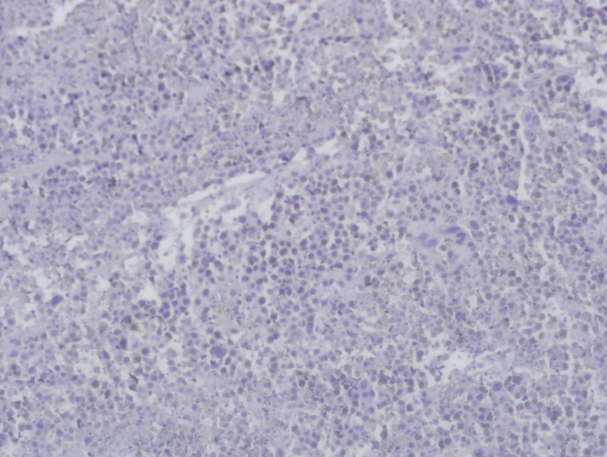

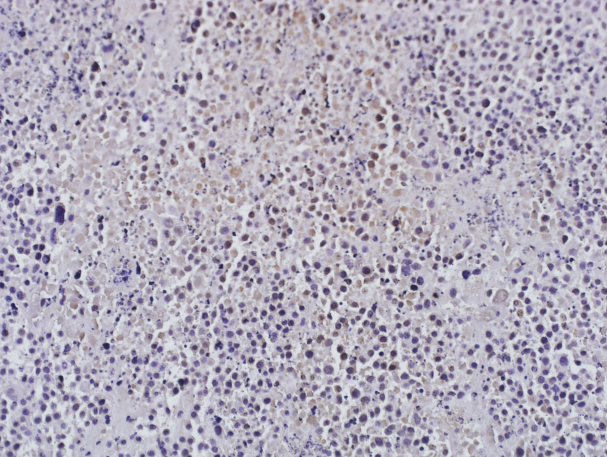


P21 si-STIM1

P21 NC


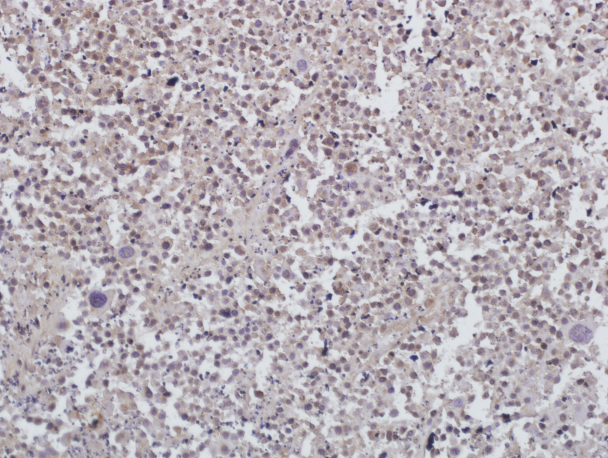

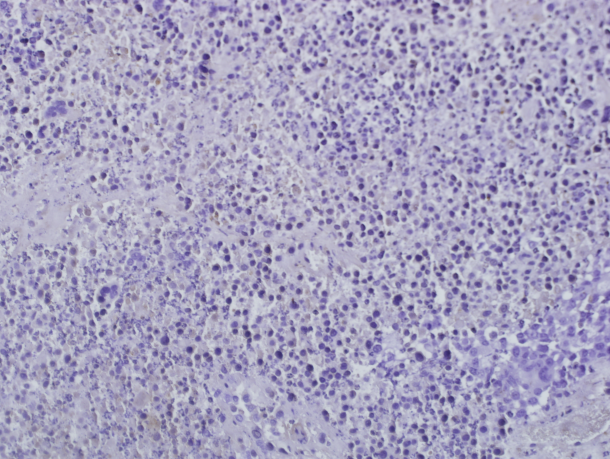


STIM1 si-STIM1

STIM1 NC


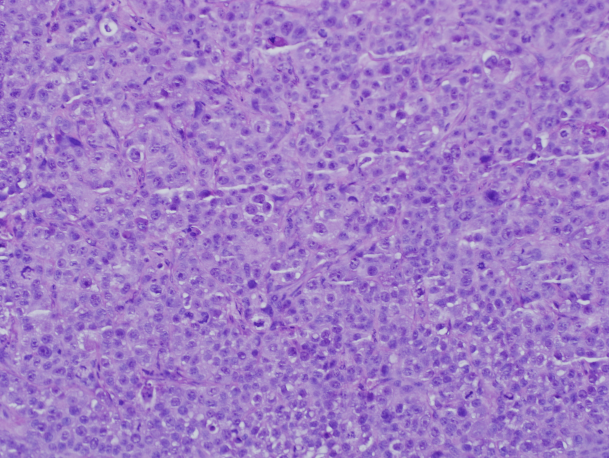

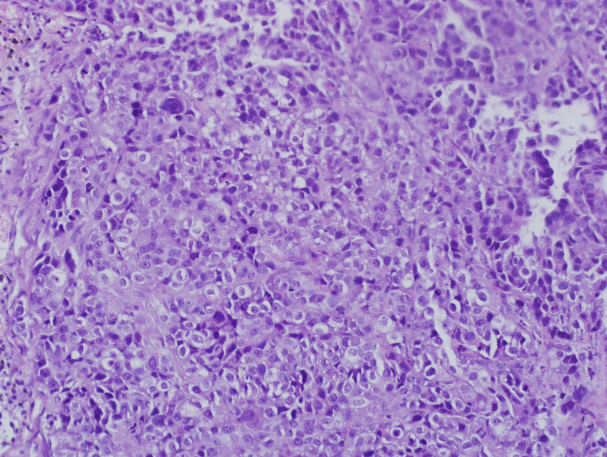


HE si-STIM1

HE NC
